# KChIP1 splice variants modulate Kv4 channels by promoting P/C-type inactivation features

**DOI:** 10.1038/s41598-026-35770-5

**Published:** 2026-01-16

**Authors:** Wuyou Cao, Georgios Tachtsidis, Robert Bähring

**Affiliations:** Institut für Zelluläre und Integrative Physiologie, Zentrum für Experimentelle Medizin, Universitätsklinikum Hamburg-Eppendorf, 20246 Hamburg, Germany

**Keywords:** A-type current, Repetitive firing, Recovery from inactivation, Exponential fitting, *Xenopus* oocytes, Two-electrode voltage-clamp, Biophysics, Neuroscience

## Abstract

Kv4 channels mediate a somatodendritic A-type potassium current, which controls neuronal excitability and firing frequency. Kv4 channels form complexes with auxiliary DPPs and KChIPs, which modify channel gating, usually including an acceleration of recovery from inactivation. Although ternary Kv4 + DPP + KChIP complexes represent a likely native channel configuration, little is known about the concerted Kv4 channel modulation by DPPs and KChIPs. Here, we studied the modulatory effects of two functionally distinct KChIP1 splice variants (1a and 1b), utilizing two-electrode voltage-clamp in *Xenopus* oocytes. We tested Kv4.1, Kv4.2, Kv4.3 S, and Kv4.3 L, co-expressed with either KChIP1 splice variant in binary Kv4 + KChIP1 and ternary Kv4 + DPP + KChIP1 channel configurations. All Kv4.x channels exhibited a second slow component of recovery from inactivation upon co-expression of either KChIP1 splice variant, which persisted in a ternary configuration with DPP. The effect was stronger for KChIP1b, suggesting a functional role of alternative splicing, by limiting fast repetitive availability of the somatodendritic A-type current. Our mechanistic investigations of ternary Kv4.2 + DPP + KChIP1b channels revealed a strong enhancement of P/C-type inactivation features, which are normally vestigial in Kv4 channels, but may co-exist with preferential closed-state inactivation in the presence of KChIP1b.

**Supplementary Information:**

The online version contains supplementary material available at 10.1038/s41598-026-35770-5.

## Introduction

Voltage-gated potassium (Kv) channels control action potential generation, shape and frequency^1^. The Kv4 channel subfamily underlies a somatodendritic A-type (i.e., rapidly inactivating) current (*I*_SA_)^2,3^, involved in the control of low frequency neuronal firing, dendritic excitation and synaptic plasticity^4–7^. Like all Kv channels, Kv4 channels are formed by the assembly of four α-subunits, surrounding a central ion conduction pathway (Fig. S1A). Each α-subunit consists of six transmembrane segments (S1–S6) with intracellular N- and C-termini and an N-terminal tetramerization (T) domain, which controls subfamily-specific assembly^8^. The transmembrane portions of the α-subunit can be divided into a voltage sensing module (S1–S4) and a pore module (S5-S6) with a selectivity filter sequence located between S5 and S6 (Fig. S1B). The distal S6 segments constitute the gate of the tetrameric channel by forming an aperture, able to either constrict or splay apart in a voltage-dependent manner^9^. Blocking of the open channel from the cytoplasmic side by an intrinsic N-terminal inactivation domain (N-type inactivation)^10^, and conformational rearrangements of the external pore mouth near the selectivity filter (P/C-type inactivation)^11^, two well-investigated mechanisms of Kv channel autoinhibition, are vestigial in Kv4 channels, which undergo preferential closed-state inactivation (CSI) based on dynamic S6 rearrangements^12–14^.

Kv4 channels form stable complexes with specific auxiliary β-subunits, including membrane-spanning dipeptidyl amino peptidase-related proteins (DPPs)^15^ and cytoplasmic Kv channel interacting proteins (KChIPs)^16^. In addition, Kv4 channels have been shown to be modulated by various proteins, including Kvβ-subunits, the K^+^ channel accessory protein (KChAP), as well as MinK and MinK-related proteins (MiRPs) belonging to the KCNE family^17,18^. Association of DPPs and KChIPs enhances Kv4 channel surface expression and modulates Kv4 channel inactivation gating in a specific manner. Typically, macroscopic inactivation of Kv4 channels is accelerated by DPPs and slowed by KChIPs, whereas recovery from inactivation is accelerated by both^15,16,19–23^. Structures of ternary Kv4 + DPP + KChIP complexes^14,24,25^, a likely native channel configuration^18^, indicate that subunit assembly occurs in a 4:4:4 stoichiometry (Fig. S1A).

There are four human KChIP subtypes (KChIP1–4), encoded by four different genes (*KCNIP1–4*)^26^. Utilizing alternative transcription start sites and alternative splicing, *KCNIP1* yields different KChIP1 isoforms. The KChIP1a and KChIP1b splice variants differ by an N-terminal 11-amino-acid stretch, rich in aromatic side chains, which is only found in KChIP1b^27,28^ (Fig. S2). This N-terminal aromatic cluster has been reported to be responsible for the differential channel modulation by KChIP1b when co-expressed by transient cDNA transfection of a stable Kv4.2-expressing cell line. In particular, KChIP1b was found to induce an extremely slow component of recovery from inactivation^28,29^, when other KChIPs, including KChIP1a, usually accelerate the recovery of Kv4 channels from inactivation^16,19,20,28^.

Here, we asked whether the peculiar KChIP1b feature of slowing recovery from inactivation is also observed in a ternary configuration with DPP and for other Kv4.x channels. Intriguingly, our data reveal the induction of a slow recovery component for the co-expression of both KChIP1a and KChIP1b (referred to as 1a and 1b for simplicity below). The effect is consistently observed for all Kv4.x channels, both in a binary configuration and in a ternary configuration with DPP. These results suggest a functional role for the KChIP1-mediated slow recovery from inactivation, which was therefore addressed mechanistically.

## Results

### Modulation of Kv4 channel-mediated A-type currents by the KChIP splice variants 1a and 1b

**Fig. 1:**
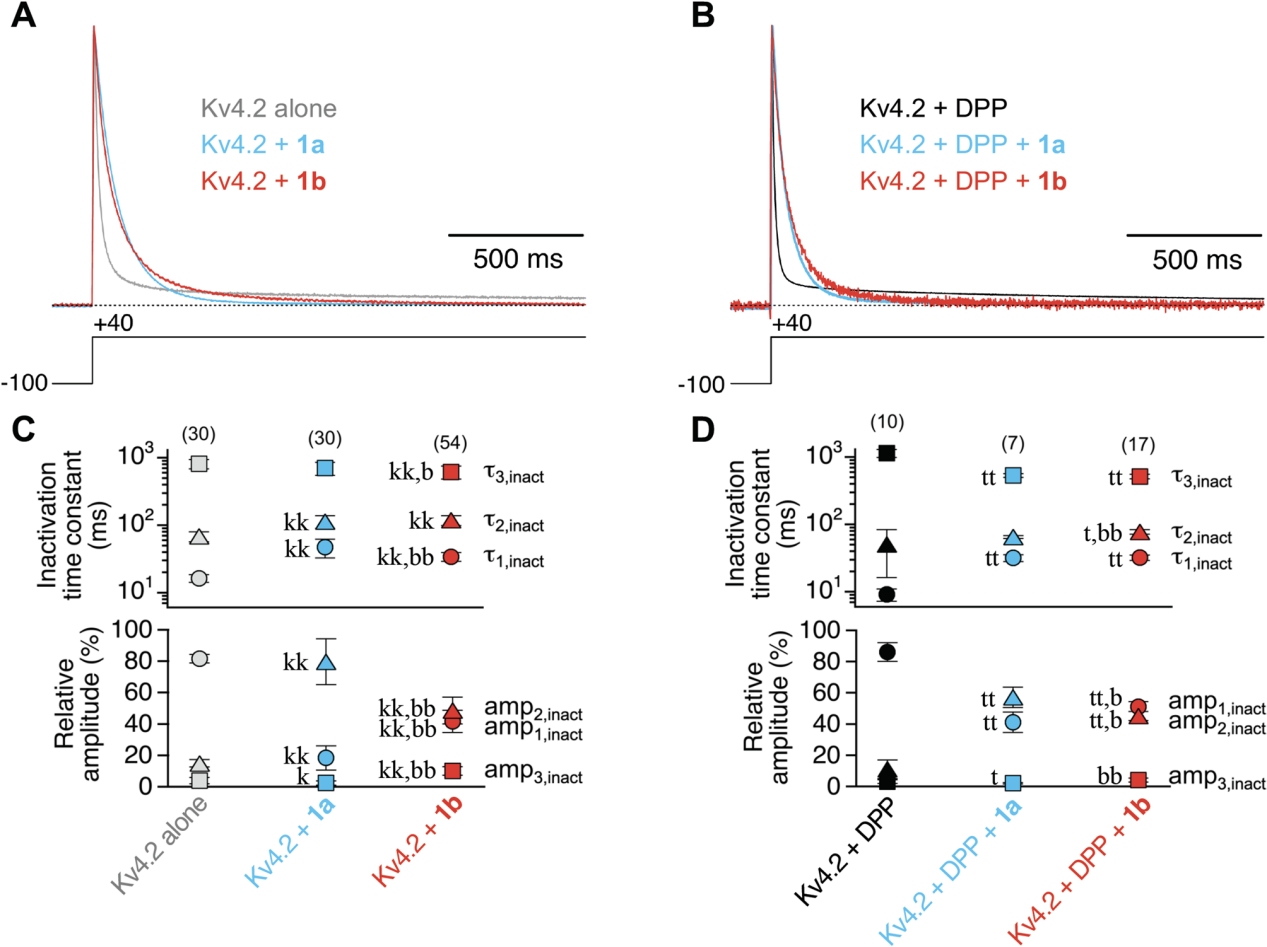
Modulation of Kv4.2 channel macroscopic inactivation by the KChIP splice variants 1a and 1b. **A**, **B** Representative current traces obtained for Kv4.2 in the absence of KChIP1 (grey, black) or co-expressed with 1a (blue) or 1b (red) in *Xenopus* oocytes in a binary configuration (A) or in a ternary configuration with DPP (B). Currents were normalized to peak and superimposed to demonstrate the effects of KChIP1 co-expression on current decay kinetics; horizontal dotted lines represent zero current. Note the typical crossover of current traces in the absence and presence of KChIP1, respectively^20^. The decay kinetics were described by the sum of three exponential functions (see Methods). **C**, **D** Inactivation time constants and their relative amplitudes (%) obtained with triple-exponential fits (see Methods); data obtained with 10 ng KChIP1 cRNA per oocyte and pooled from different days after cRNA injection; number of oocytes (n) indicated. Lower case letters denote significant differences; single letter: *p* < 0.05; two letters: *p* < 0.0001; k, kk: significantly different from Kv4.2 alone; t, tt: significantly different from Kv4.2 + DPP; b, bb: significantly different from co-expression with 1a (see also Table S2).

**Fig. 2:**
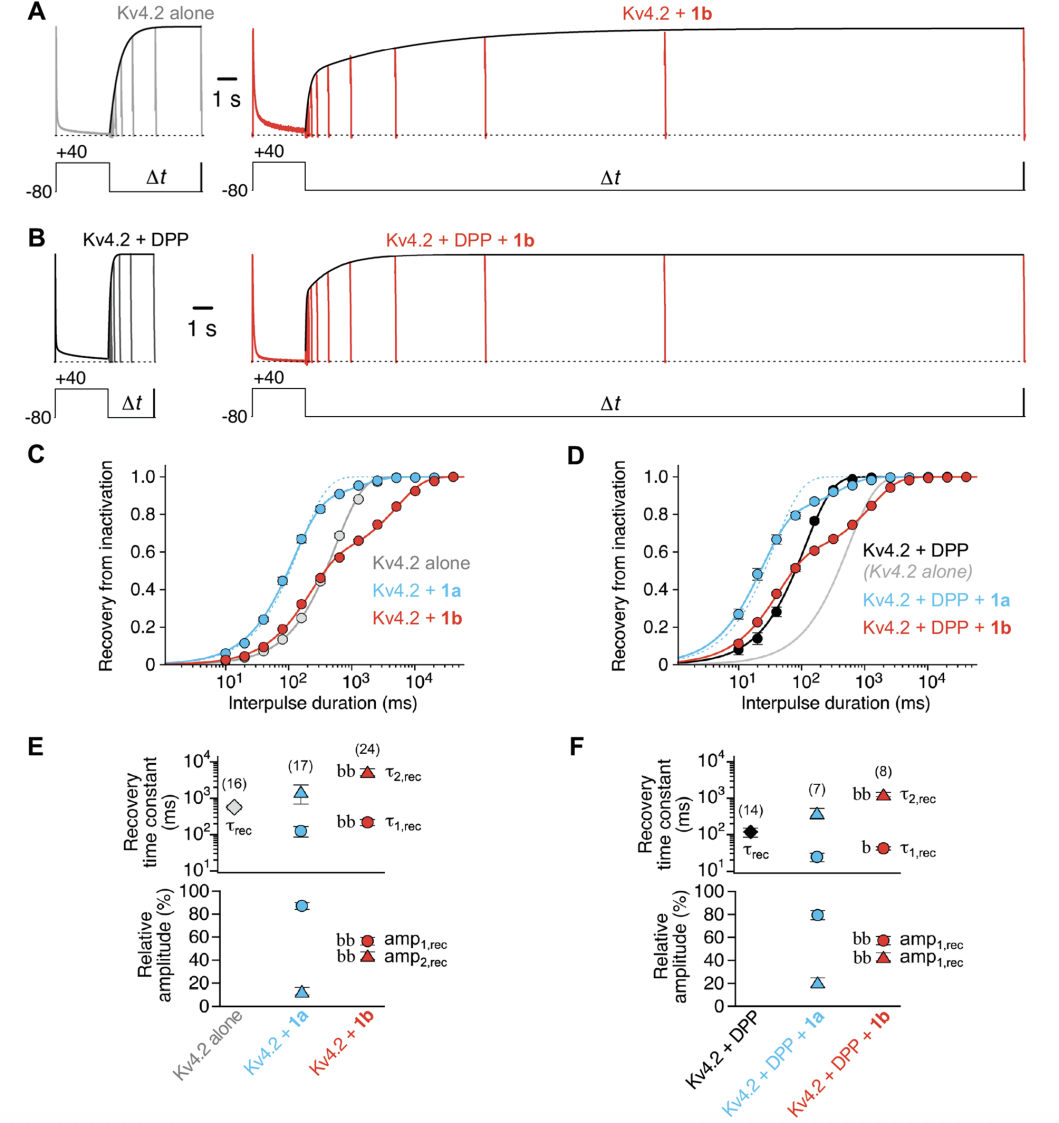
Modulation of Kv4.2 channel recovery kinetics by the KChIP splice variants 1a and 1b. **A**, **B** Recordings obtained with a recovery protocol from oocytes expressing Kv4.2 alone (A, left; grey), Kv4.2 + 1b (A, right; red), Kv4.2 + DPP (B, left; black) or Kv4.2 + DPP + 1b (B, right; red); control current amplitudes were normalized; horizontal dotted lines represent zero current. **C**, **D** Relative current amplitudes were plotted against interpulse duration on a log-scale, and the data were described by exponential functions (see Methods and enveloping curves along the test current peaks in A and B). Dotted blue lines in C and D: single-exponential function applied to the data obtained with 1a co-expression; grey line without symbols in D: fitting curve obtained for Kv4.2 alone (C). **E**, **F** Recovery time constants including their relative amplitudes (%); data obtained with 10 ng KChIP1 cRNA per oocyte and pooled from different days after cRNA injection; number of oocytes (n) indicated. Lower case letters denote significant differences; single letter: *p* < 0.05; two letters: *p* < 0.0001; k, kk: significantly different from Kv4.2 alone; t, tt: significantly different from Kv4.2 + DPP; b, bb: significantly different from co-expression with 1a (see also Table S2).

**Fig. 3:**
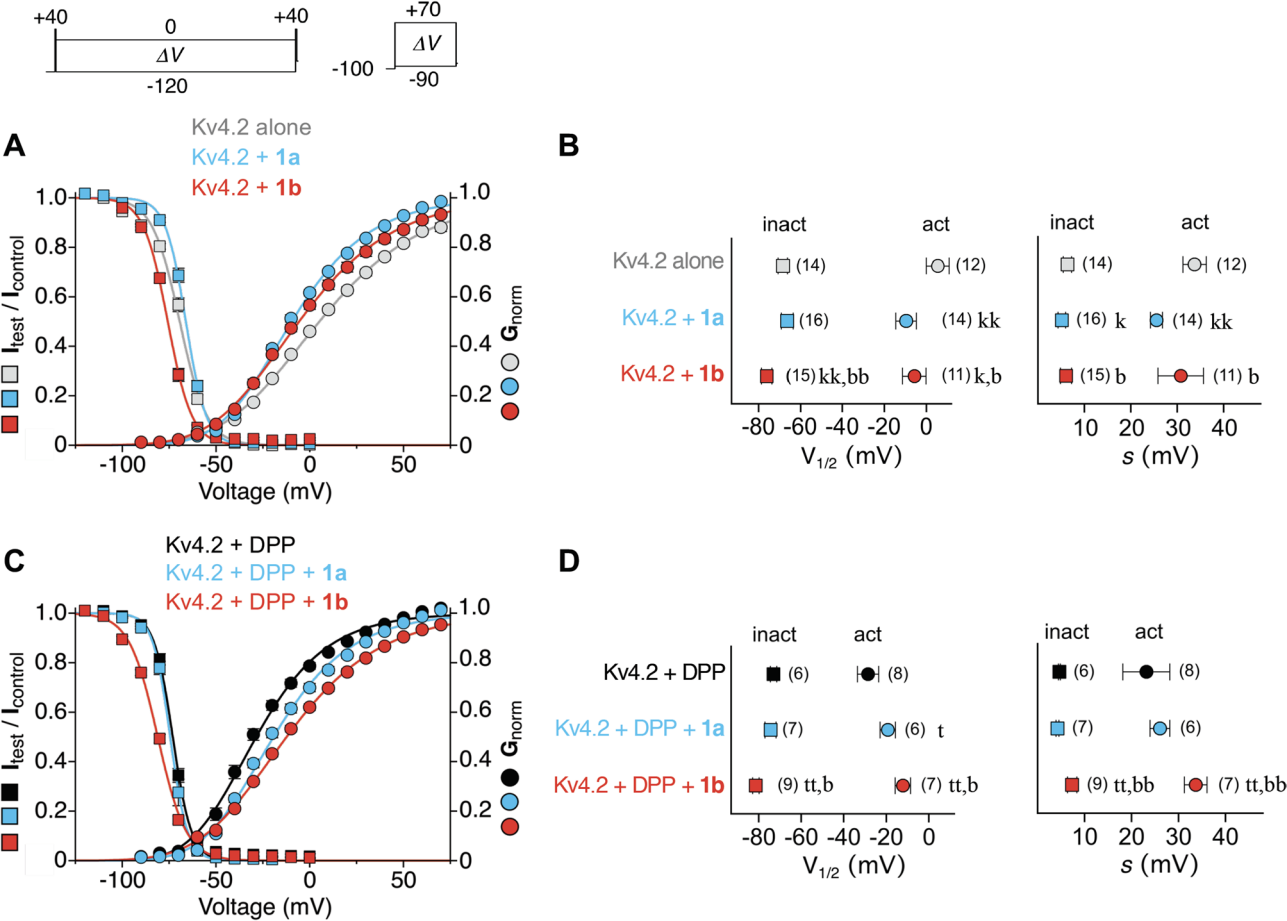
Effects of KChIP splice variants 1a and 1b on the voltage dependence of Kv4.2 channel gating. Raw data not shown, but see insets in the upper left for voltage protocols. **A**,** C** Normalized data from steady-state inactivation protocols and normalized conductance values (G_norm_) from activation protocols obtained for Kv4.2 in the absence of KChIP1 (grey, black) or co-expressed with 1a (blue) or 1b (red) in a binary configuration (A) or in a ternary configuration with DPP (C). The data were plotted against conditioning or test pulse voltage, respectively, in the same graph and fitted with appropriate Boltzmann-functions^13^ (see Methods). **B**,** D** Voltages of halfmaximal activation (act, circles) and inactivation (inact, squares) and corresponding slope factors (V_1/2_ and s, respectively); data obtained with 10 ng KChIP1 cRNA per oocyte and pooled from different days after cRNA injection; number of oocytes (n) indicated. Lower case letters denote significant differences; single letter: *p* < 0.05; two letters: *p* < 0.0001; k, kk: significantly different from Kv4.2 alone; t, tt: significantly different from Kv4.2 + DPP; b, bb: significantly different from co-expression with 1a (see also Table S2).

To study different aspects of Kv4 channel modulation by the two KChIP splice variants 1a and 1b, we expressed Kv4.x channels (Kv4.1, Kv4.2, Kv4.3 S or Kv4.3 L) in the following configurations: (1) Kv4.x α-subunit alone; (2) Kv4.x α-subunit together with either 1a or 1b; (3) Kv4.x α-subunit together with DPP; (4) Kv4.x α-subunit together with DPP and either 1a or 1b (Fig. S1). The effects of 1a or 1b co-expression on the following parameters were examined: (1) Macroscopic inactivation kinetics; (2) Kinetics of recovery from inactivation; (3) Voltage dependences of activation and steady-state inactivation (see Methods). The present study was primarily focussed on the effects of KChIP1 splice variant co-expression on Kv4.2 (Figs. 1, 2 and 3), in order to complement previously published data obtained with a Kv4.2 stable Human Embryonic Kidney (HEK) 293 cell line, transiently transfected with 1a or 1b cDNA in the absence of DPP^28,29^. In the Supplements further experimental data for Kv4.2 and the results obtained for Kv4.1, Kv4.3 S and Kv4.3 L are illustrated (Figs. S3–S7), and the data are summarized (Tables S1–S5).

We initially analysed the kinetics of macroscopic inactivation (Fig. 1A, B), and obtained time constants of ∼ 16, 68, 809 ms (81, 15, 4%) for Kv4.2 alone and ∼ 9, 50, 1136 ms (86, 11, 3%) for Kv4.2 + DPP (Fig. 1C, D). Co-expression of KChIP1 splice variants caused the typical crossover of normalized current traces when overlayed with control traces obtained in the absence of KChIP1^20^. This was due to a slowing of the initial and intermediate decay components, while the final decay component was unaffected or slightly accelerated by KChIP1 co-expression. The corresponding time constants were ∼ 47, 113, 697 ms (18, 80, 2%) for Kv4.2 + 1a, and ∼ 34, 118, 615 ms (42, 48, 10%) for Kv4.2 + 1b (Fig. 1C; see also Fig. S3A and Table S2). In the ternary configuration we obtained time constants of ∼ 32, 65, 531 ms (41, 57, 2%) for Kv4.2 + DPP + 1a, and ∼ 33, 85, 483 ms (48, 48, 4%) for Kv4.2 + DPP + 1b (Fig. 1D; Table S2). The effects of auxiliary subunit co-expression on Kv4.1, Kv4.3 S and Kv4.3 L-mediated current decay kinetics showed a considerable variability, but the typical acceleration of the final cumulative component of macroscopic inactivation was consistently observed for both 1a and 1b co-expression and in either channel configuration (Fig. S4; Tables S1, S3 and S4). Thus, our data show that both KChIP1 splice variants exert the usual effects on current decay kinetics^20^.

### Unusual modulation of Kv4 channel recovery kinetics by the KChIP splice variants 1a and 1b

In the absence of KChIP1 the recovery of Kv4.2 channels from inactivation followed a single-exponential time course, and the recovery kinetics were accelerated by DPP co-expression^15^ (Fig. 2A–D). The recovery time constants were 563 ms for Kv4.2 alone and 117 ms for Kv4.2 + DPP (Fig. 2E, F; Table S2). It has been reported previously that, unlike 1a, 1b causes biphasic kinetics of recovery from inactivation, resulting in a slowing of the overall recovery process in Kv4.2 channels^28,29^. Surprisingly, we observed biphasic recovery kinetics for both 1a and 1b co-expression in either channel configuration (Fig. 2C, D), albeit the obtained time constants and their relative amplitudes differed for the two KChIP1 splice variants, with a more pronounced slowing for 1b. In the binary configuration, we obtained recovery time constants of 126 ms and ∼1.5 s (87 and 13%) for Kv4.2 + 1a, and 218 ms and ∼5.4 s, (56 and 44%) for Kv4.2 + 1b. Of note, the distinct remodelling features seen for 1a and 1b co-expression in a binary configuration did not depend on the extent of KChIP1 overexpression (Fig. S3B; Table S2). In a ternary configuration, the recovery time constants were ∼24 and 395 ms (79 and 21%) for Kv4.2 + DPP + 1a, and ∼ 41 ms and 1.3 s (57 and 43%) for Kv4.2 + DPP + 1b (Fig. 2F; Table S2). The modulation of recovery kinetics caused by 1a and 1b co-expression observed for Kv4.1, Kv4.3 S and Kv4.3 L in a binary and a ternary configuration was very similar to the results obtained with Kv4.2 (Fig. S5; Tables S1, S3 and S4). Thus, although inconspicuous regarding the modulation of current decay kinetics, KChIP1 splice variant co-expression effects on recovery kinetics, especially for 1b, markedly differ from the usual KChIP effects^16,19,20^ in all channel configurations tested.

### Voltage dependence of Kv4 channel steady-state inactivation is differentially modulated by KChIP splice variants 1a and 1b

Finally, we examined the effects of 1a and 1b co-expression on the voltage dependences of activation and steady-state inactivation (Fig. 3). For all channel configurations tested, the Kv4.2 activation and inactivation curves showed little overlap, characteristic for preferential CSI^12^, and inactivation curves were steeper than activation curves (Fig. 3A, C). For Kv4.2 alone, halfmaximal activation occurred at + 5.5 mV with a slope factor of 33.8 mV. DPP co-expression resulted in a negative shift of the activation curve^15^, with halfmaximal activation at − 28.5 mV (slope factor 23.2 mV) for Kv4.2 + DPP (Fig. 3B, D). In a binary configuration, co-expression of KChIP1 splice variants caused a steepening and a negative shift of activation curves with halfmaximal activation at − 9.6 mV (slope factor 25.6 mV) and − 5.7 mV (slope factor 30.8 mV) for Kv4.2 + 1a and Kv4.2 + 1b, respectively (Fig. 3A, B). In a ternary configuration with DPP, KChIP1 co-expression caused a positive shift of activation curves, with halfmaximal activation at − 19.2 mV (slope factor 26.1 mV) and − 12.1 mV (slope factor 33.7 mV) for Kv4.2 + DPP + 1a and Kv4.2 + DPP + 1b, respectively (Fig. 3C, D; see also Table S2).

The analysis of steady-state inactivation revealed that halfmaximal inactivation occurred at − 68.3 mV (slope factor 6.4 mV) for Kv4.2 alone. Similar to the voltage dependence of activation, the inactivation curve was shifted negative by DPP co-expression^15^, with halfmaximal inactivation at − 73.5 mV (slope factor 4.7 mV) for Kv4.2 + DPP (Fig. 3B, D). The modulation of the voltage dependence of steady-state inactivation differed between 1a and 1b co-expression. While 1a caused virtually no effect, 1b shifted inactivation curves in the negative direction (Fig. 3A, C). In a binary configuration, halfmaximal inactivation occurred at − 66.3 mV (slope factor 5.2 mV) for Kv4.2 + 1a and at − 76.0 mV (slope factor 6.1 mV) for Kv4.2 + 1b (Fig. 3B; see also Fig. S3C and Table S2). In a ternary configuration with DPP, halfmaximal inactivation occurred at − 74.4 mV (slope factor 4.3 mV) for Kv4.2 + DPP + 1a and at − 81.3 mV (slope factor 7.4 mV) for Kv4.2 + DPP + 1b (Fig. 3D; Table S2). The modulation of the voltage dependences of Kv4.1, Kv4.3 S and Kv4.3 L gating caused by 1a and 1b co-expression in a binary and a ternary configuration was very similar to the results described for Kv4.2. Although the results showed some variability among the different Kv4.x channels, binary and ternary channels containing 1b had the most negative inactivation curves (Fig. S6; Tables S1, S3 and S4).

### High external K^+^ identifies P/C-type inactivation features of the 1b-mediated slow recovery component

Our kinetic analyses of recovery from inactivation have corroborated the view that the biphasic nature of the recovery process represents an intrinsic feature of KChIP1-containing Kv4 channels. Moreover, our data suggest that, unlike 1a, 1b co-expression induces only negligible acceleration of recovery, but more or less exclusively adds a slow recovery component (see Fig. 2C, D). Therefore, we chose Kv4.2 + DPP + 1b ternary channels to study the kinetics of recovery from inactivation in more mechanistic detail. To this end, we intended to experimentally interfere with different mechanisms of inactivation, normally vestigial in Kv4 channels^12^, but possibly promoted by 1b co-expression, such as N-type inactivation^30^ or P/C-type inactivation^31^.

**Fig. 4:**
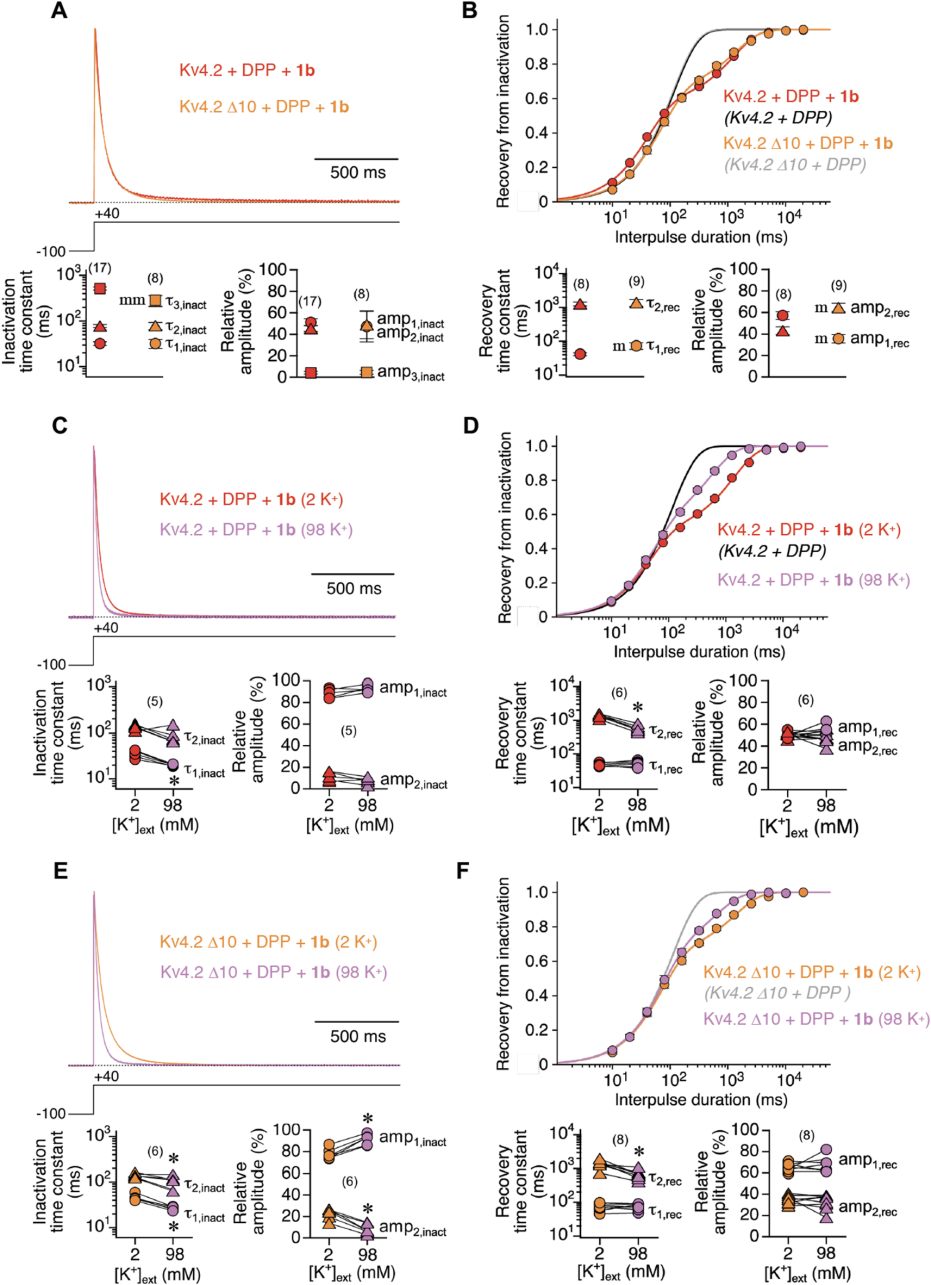
Macroscopic inactivation and recovery kinetics of ternary Kv4.2 + DPP + 1b channels after N-terminal truncation and in the presence of elevated external K^+^. **A**, **C**,** E** The kinetics of macroscopic inactivation were analysed for wild-type (red) and N-terminally truncated (Δ10, orange) ternary Kv4.2 channels in standard ND96 (2 K^+^) and high external K^+^ solution (98 K^+^, purple; see Methods). Currents were normalized to peak and superimposed; horizontal dotted lines represent zero current. Inactivation time constants and their relative amplitudes (%) are shown below. Note that current decay kinetics were approximated by a double-exponential function in the solution exchange experiments (C and E). **B**, **D**, **F** Recovery plots including double-exponential fits for wild-type and N-terminally truncated channels in standard ND96 and high external K^+^ solution. Congruent single-exponential fitting curves (black and grey) without symbols: Kv4.2 + DPP and Kv4.2 Δ10 + DPP. Recovery time constants and their relative amplitudes (%) are shown below. Data pairs of solution exchange experiments in C, D, E and F and number of oocytes (n) indicated. Lower case letters and asteriscs denote significant differences; single letter or asterisc: *p* < 0.05; two letters: *p* < 0.0001; m, mm: significantly different from Kv4.2 wild-type; *: significantly different from 2 K^+^ (see also Table S5).

Vestigial N-type inactivation features of Kv4 channels are thought to be largely suppressed by KChIP binding^30^. Nevertheless, in order to abolish putative residual or newly generated N-type inactivation features in Kv4.2 + DPP + 1b channels, we used an N-terminally truncated version of Kv4.2, which lacks the first 10 amino acids (Δ10; Fig. S1B). This deletion removes the Kv4.2 N-terminal inactivation domain, but leaves KChIP binding intact^19,30,32^. We found that macroscopic inactivation kinetics of the truncated channels were almost identical to wild-type (Fig. 4A). Also, the kinetics of recovery from inactivation were still biphasic and very similar to wild type (Fig. 4B; Table S5). Based on these results, residual or newly generated N-type inactivation as a possible mechanism underlying the slow recovery kinetics of 1b containing Kv4.2 channel complexes was excluded.

Next, we intended to interfere with putative residual or newly generated P/C-type inactivation in Kv4.2 + DPP + 1b channels by applying a high K^+^ solution (see Methods). Elevated external K^+^ has been shown previously to slow current decay and to accelerate recovery kinetics by interfering with the classical P/C-type inactivation of *Shaker*-related (Kv1) channels^33–37^. Under these conditions, the macroscopic inactivation of Kv4.2 + DPP + 1b channels was accelerated, in accordance with previous reports^31,38–41^ (Fig. 4C; see Discussion). Intriguingly, high K^+^ solution also affected the kinetics of recovery from inactivation by specifically accelerating the slow component (2.4-fold; Fig. 4D; Table S5). High K^+^ solution also influenced Kv4.2 Δ10 + DPP + 1b channels, in the same manner as wild-type ternary channels, albeit with a somewhat weaker effect on the slow recovery component (2.1-fold acceleration; Fig. 4E, F). Notably, superfusion with TEA solution (see Methods) had no obvious effects, except for an acceleration of current decay kinetics, which was more pronounced for Kv4.2 Δ10 than for Kv4.2 wild-type ternary channels (Fig. S7; see also Table S5).

**Fig. 5:**
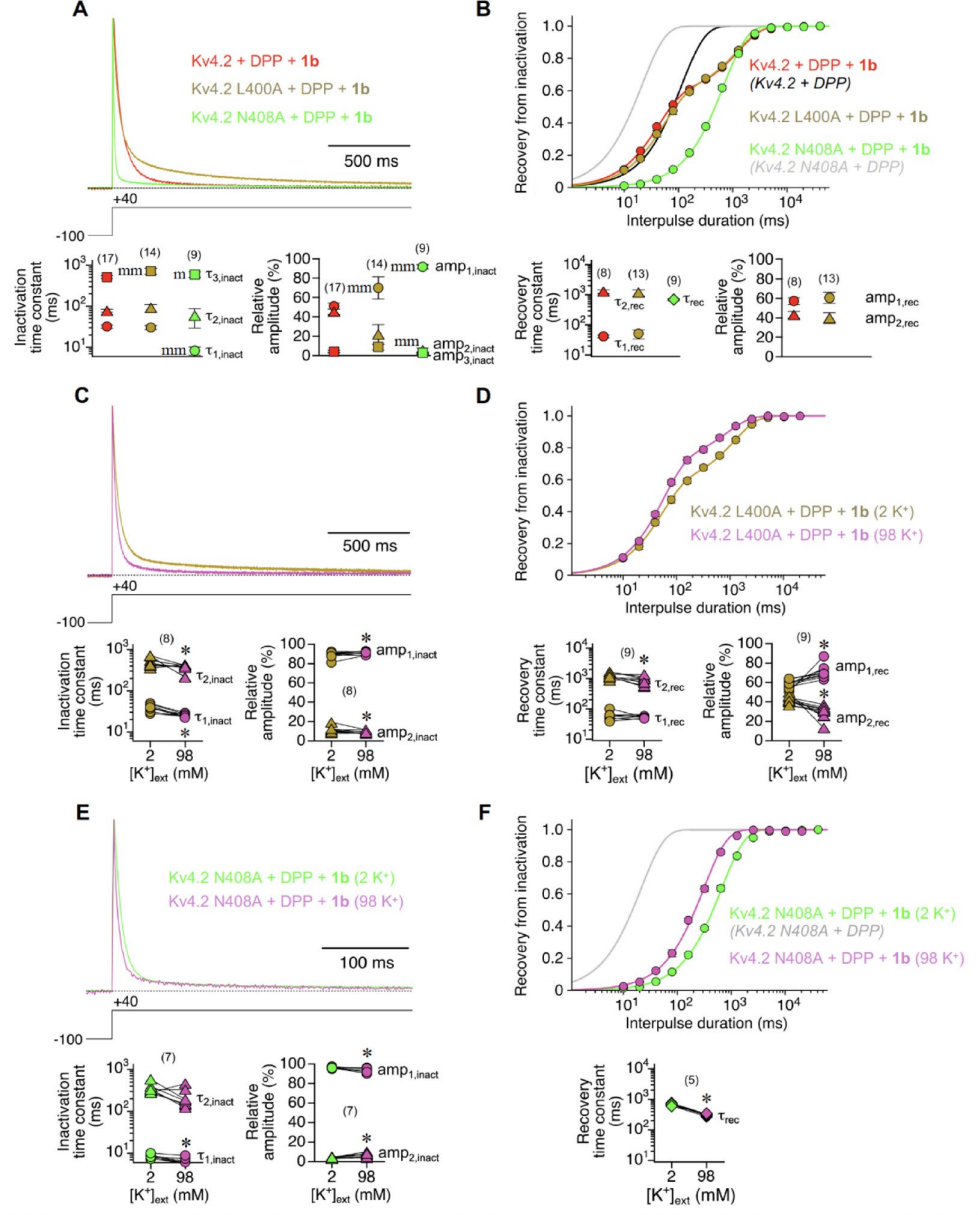
K^+^ dependence of macroscopic inactivation and recovery kinetics in ternary Kv4.2 + DPP + 1b channels with mutations in S6. A The kinetics of macroscopic inactivation were analysed for wild-type Kv4.2 channels (red) and Kv4.2 channels with amino acid exchanges in S6 (L400A: bronze; N408A: green) in a ternary configuration. **C**,** E** Effects of high external K^+^ solution (98 K^+^, purple; see Methods) on the macroscopic inactivation kinetics of L400A (C) and N408A (E) ternary Kv4.2 channels. In A, C and E currents were normalized to peak and superimposed, and horizontal dotted lines represent zero current. Inactivation time constants and their relative amplitudes (%) are shown below. Note the expanded time scale in E, and that current decay kinetics were approximated by a double-exponential function in the solution exchange experiments (C and E). **B** Recovery plot including exponential fits for Kv4.2 wild-type, L400A and N408A ternary Kv4.2 channels. **D**,** F** Effects of high external K^+^ on the recovery kinetics of L400A (D) and N408A (F) ternary Kv4.2 channels. Recovery time constants and their relative amplitudes (%) are shown below. Note that N408A ternary Kv4.2 channel recovery kinetics followed a single-exponential time course. Single-exponential fitting curves without symbols: Kv4.2 + DPP (black) and Kv4.2 N408A + DPP (grey) in standard ND96. Data pairs of solution exchange experiments in C, D, E and F and number of oocytes (n) indicated. Lower case letters and asteriscs denote significant differences; single letter or asterisc: *p* < 0.05; two letters: *p* < 0.0001; m, mm: significantly different from Kv4.2 wild-type; *: significantly different from 2 K^+^ (see also Table S5).

From our experimental results with high K^+^ solution we concluded that Kv4.2 + DPP + 1b channels may not only undergo CSI related to dynamic S6 rearrangements^13,14^, from which recovery is thought to be fast. Rather, promoted by 1b co-expression, a fraction of channels may also undergo strong *Shaker*-like P/C type inactivation with slow recovery kinetics. Therefore, we finally intended to study Kv4.2 channel constructs in which S6-related CSI was specifically modified. For this purpose, we chose two previously characterized S6 mutants (Fig. S1B), in which recovery from inactivation had been found to be either drastically slowed (Kv4.2 L400A) or drastically accelerated (Kv4.2 N408A)^13^. We expected the putative newly generated P/C-type inactivation with slow recovery kinetics in the presence of 1b to be further augmented in Kv4.2 N408A, where the affinity towards S6-related CSI states is lowered^13^ (see Discussion), but not in Kv4.2 L400A. The S6 mutations influenced both macroscopic inactivation and recovery from inactivation in Kv4.2 + DPP + 1b channels in a characteristic manner. While L400A caused an overall slowing, N408A caused a strong acceleration of current decay kinetics (Fig. 5A). Kv4.2 L400A + DPP + 1b recovery kinetics were very similar to wild-type ternary, whereas Kv4.2 N408A + DPP + 1b recovery kinetics differed substantially: They followed a single exponential time course, apparently corresponding to the slow recovery components of wild-type and L400A ternary channels (Fig. 5B; see also Table S5). Finally, we tested the two mutants in high K^+^ solution. For both mutants, macroscopic inactivation was accelerated under these conditions, similar to wild type (Fig. 5C, E; see also Fig. 4C). Also, the slow recovery component was accelerated in L400A ternary channels, as seen for wild-type, albeit only 1.4-fold (Fig. 5D). Remarkably, in N408A ternary channels the recovery kinetics remained single-exponential, and were accelerated 2.2-fold in high K^+^ solution (Fig. 5F; see also Table S5), suggesting that Kv4.2 N408A + DPP + 1b recovery kinetics fully reflect the recovery from a putative *Shaker*-like P/C type inactivation. Taken together, our findings support the notion that otherwise vestigial P/C-type inactivation features of Kv4.2 channels are strongly promoted by 1b and may co-exist with S6-related CSI.

## Discussion

In the present paper we set out to critically revise a previous report on the distinctive features of the KChIP splice variants 1a and 1b^28^. The previously reported induction of a novel extremely slow component of recovery from inactivation by 1b is confirmed by our results, but they reveal that 1a co-expression can also cause biphasic recovery kinetics. KChIP1 co-expression effects in heterologous systems and the putative mechanism, including possible structure-function relationships, underlying the slow recovery component, will be discussed in a physiological context.

Co-expression of an initially identified KChIP1, which is identical to the 1a splice variant used herein, with Kv4.2 in tissue culture cells and *Xenopus* oocytes defined the hallmarks of Kv4 channel modulation by KChIPs. These included an increase in current density, a slowing of macroscopic inactivation and an acceleration of recovery from inactivation^16^. The initially identified KChIP1 (= 1a) was used for a detailed analysis of Kv4 channel assembly and trafficking, to demonstrate the stabilizing effect of KChIP1 on Kv4.3 tetramers, as well as the KChIP1-mediated release of Kv4.2 ER retention, and the role of KChIP1-specific N-terminal myristoylation in subcellular targeting of the Kv4.2 + KChIP1 complex to post-ER transport vesicles^22,42–44^. These results provided an explanation for the observed increase in current density upon KChIP1 co-expression. The initially identified KChIP1 (= 1a) was also used for a detailed biophysical analysis of Kv4 channel gating modulation, showing similar remodelling of Kv4.1 and Kv4.3 channel inactivation^20^. The remodelling included the typical streamlining effect on macroscopic currents, with a crossover of normalized current traces obtained in the absence and presence of KChIP1, respectively (see also Fig. 1 and Fig. S4), as well as a shift of inactivation curves to more positive voltages, most likely reflecting the accelerated recovery from inactivation, upon KChIP1 co-expression^20^. Our experimental results obtained with Kv4.x + 1a co-expression largely confirm these initial observations.

The alternatively spliced KChIP variant 1b differs from 1a by an 11-amino-acid N-terminal insertion (residues 22–33 in 1b; Fig. S2), rich in aromatic side chains^26–28^. The 1b splice variant has been reported previously to differ from 1a quite substantially, by inducing biphasic kinetics of recovery from inactivation with a newly generated extremely slow component^28^. Our critical revision of this previous report on the distinctive features of 1b was motivated by the fact that the authors had used transient transfection of a stable Kv4.2 cell-line with KChIP1 cDNA. We suspected that in this system, cell-to-cell variations in cDNA uptake and differences in the temporal expression profiles of α- (stable) and β-subunits (transient), may have caused two different Kv4.2 channel populations present at roughly equal amounts in the plasma membrane; i.e., Kv4.2 + 1b and Kv4.2 alone (stable), with fast and slow recovery kinetics, respectively. Therefore, we subjected the previous findings to a rigorous test by studying KChIP1 co-expression effects on Kv4.2 channel gating in a more quantitative manner in cRNA-injected *Xenopus* oocytes. With this approach the previously reported biphasic recovery kinetics were confirmed, irrespective of the amount of 1b cRNA injected (see Fig. 2 and Fig. S3B), excluding with some certainty the possibility that channels not occupied by KChIP1 may have caused slow recovery kinetics. Thus, our initial concerns regarding the previously used expression system and transfection procedure were clearly unfounded. We further asked whether the 1b effect on recovery kinetics is also observed with other Kv4.x subtypes, and in a ternary configuration with DPP, which itself strongly accelerates the recovery of Kv4 channels from inactivation^15^, possibly masking the 1b effect. This approach also included the two Kv4.3 splice variants S and L, which have been previously shown to differ with respect to KChIP2 modulation, albeit not in terms of recovery kinetics^45^. In fact, we found that Kv4.3 L + 1b channels exhibit slower biphasic recovery kinetics than Kv4.3 S + 1b channels, however, this difference vanished in a ternary configuration with DPP (see Tables S3 and S4). In summary, the results of our experiments performed with all Kv4.x channel subtypes co-expressed with excess amounts of 1b, both in a binary configuration and in a ternary configuration with DPP, support the notion that the induction of a slow recovery component represents an intrinsic feature of the KChIP splice variant 1b with general applicability. It should be noted, that the functional diversity caused by alternative splicing of KChIP1 may be further increased by other auxiliary proteins, especially the ones belonging to the KCNE family^45^, which was not examined in the present study.

Unexpectedly, we found that, similar to 1b, 1a is also capable of inducing a slow recovery component. It is remarkable that, for the most part, previous reports of KChIP1 (= 1a) co-expression effects on Kv4 channels, in both cDNA-transfected tissue culture cells^16,28,29,46–49^ and cRNA-injected *Xenopus* oocytes^15,16,20,24,27,31,50–55^, used a single-exponential function to describe the kinetics of recovery from inactivation. Also, previous reports on the recovery kinetics of ternary Kv4.2 + DPP + 1a channels in different expression systems^15,46^ used a single-exponential function for their analyses. Multi-exponential fitting of the 1a-induced recovery kinetics was not considered in these previous studies, presumably due to a relatively small fractional amplitude of the slow component. Also, in some of the previously used experimental protocols the chosen interpulse durations may have been not long enough to reliably capture such a slow recovery component. Notably, however, in one study, performed in *Xenopus* oocytes with 2 mM standard external K^+^, like in the present study, the initially identified KChIP1 (= 1a) was reported to cause biphasic recovery kinetics for Kv4.1 and Kv4.2^56^. Double-exponential fitting in that study resulted in a numerical ratio of fast and slow recovery time constants and in fractional amplitudes very similar to our results. Van Hoorick and co-workers were able to convert biphasic into virtually monophasic recovery kinetics by mutating three of the five aromatic amino acid residues in 1b to alanine^29^. Our kinetic analyses suggest that additional structural determinants in KChIP1 may contribute to the special remodelling features.

We have chosen Kv4.2 + DPP + 1b ternary channels to study the newly generated slow recovery component mechanistically. In addition to CSI, which is most prominent in Kv4 channels^12^, Kv channels may undergo N-type inactivation and/or P/C-type inactivation, which are the major inactivation mechanisms of *Shaker*-related (Kv1) channels^10,11^. These classical *Shaker* inactivation mechanisms are also present in Kv4 channels, but in vestigial forms^12,30,31^. Commonly used approaches; i.e., N-terminal truncation and application of high external K^+^, respectively, were applied in the present study to test for a possible enhancement of the classical *Shaker* inactivation mechanisms in Kv4 channels, caused by the KChIP splice variant 1b. Since KChIP binding is thought to sequester and immobilize a Kv4 N-terminal inactivation domain^14,24,25,30,49,52,54^, an involvement of N-type inactivation seemed rather unlikely beforehand. The virtual absence of an effect of a ten amino acid N-terminal truncation (Δ10) on Kv4.2 + DPP + 1b recovery kinetics confirmed this a priori assumption.

Application of high external K^+^ is known to slow current decay for *Shaker*-related (Kv1) channels, by a suppression of their P/C-type inactivation^35^. In non-*Shaker* channels, void of a prominent P/C-type inactivation but undergoing preferential CSI (Kv2, Kv3 and Kv4), high external K^+^ often causes an acceleration of macroscopic current decay^31,57,58^. Very similar to the results obtained by Kaulin and co-workers for Kv4.3 + KChIP1 channels^31^, high external K^+^ caused an acceleration of current decay kinetics in our study, an indication of the removal of vestigial P/C-type inactivation to favor CSI in ternary Kv4.2 + DPP + 1b channels. Remarkably, however, high external K^+^ also accelerated the slow component of the biphasic recovery process of wild-type and Δ10, and the monophasic recovery process of N408A ternary channels, reminiscent of the accelerated recovery kinetics observed previously under these conditions for the *Shaker*-related channels Kv1.3^33,34^ and Kv1.4^36,37^. The striking similarity of these high K^+^ effects on recovery kinetics supports the notion that the slow recovery component observed in Kv4 channels upon 1b co-expression is related to a *Shaker*-like P/C-type inactivation. Notably, our results suggest that 1b-independent vestigial P/C-type inactivation^31^ and a more stable 1b-induced *Shaker*-like P/C-type inactivation may co-exist in Kv4 channels. This notion is also supported by our finding that TEA influenced current decay kinetics similar to high K^+^, but left Kv4.2 + DPP + 1b recovery kinetics largely unaffected (see Fig. S7).

Apart from an immobilization of the Kv4 N-terminal inactivation domain caused by KChIP binding^14,24,25,30,49,52,54^, the structure-function relationships of Kv4 channel remodelling by KChIPs are largely unknown. In particular, the 11-amino-acid aromatic cluster, thought to be responsible for the special Kv4 remodelling by 1b^29^, is not included in the available Kv4/KChIP1 structures^24,25,49,52–54^ (Fig. S2). Thus, a putative direct interaction between the aromatic cluster and the Kv4 α-subunit remains uncertain. One may speculate whether the clamping conformation adopted by the four KChIP1 molecules surrounding the gating-relevant^59,60^ Kv4 T-domains^24,25,49,52–54^ may be influenced by the aromatic cluster. It is intriguing in this context that a naturally occurring Kv1.5 Δ209 N-terminal truncation variant, which has lost its T-domain, exhibits, in addition to classical P/C-type inactivation, a form of CSI, resulting in biphasic kinetics of recovery from inactivation^61^.

Recently high resolution cryo EM data have elucidated structural details of Kv1.2 P/C-type inactivation. The data suggest that P/C-type inactivation in these channels leads to a dilation rather than a constriction of the selectivity filter^62^, and that an isoleucine gate localized in S6, right below the selectivity, filter plays a central role^63^. Cryo EM data have also elucidated structural details of Kv4.2 channel inactivation^14^, by capturing, in addition to an open state, two putative non-conducting states, referred to as “inactivated” and “intermediate”. In both non-conducting states, an upper and a lower gate within the pore are expected to prevent the passage of K^+^ ions. The lower gate is related to the dynamic coupling between voltage sensor and pore modules, allowing conformational rearrangements, that lead to a symmetry breakdown of S6-segments as the basis of CSI^13,14^. Intriguingly, the upper gate in Kv4.2 is homologous to the isoleucine gate, which mediates P/C-type inactivation in Kv1.2^63^. Based on these structural similarities, one may speculate that in the presence of the KChIP splice variant 1b, the upper (isoleucine) gate in Kv4.2 may evolve into a major inactivation gate, especially if S6-related CSI (lower gate) is unstable, as suggested by our experimental findings with the Kv4.2 S6 mutant N408A (see Fig. 5). Of note, a close inspection of the structures put forward by Kise and co-workers^24^ and by Ma and co-workers^25^, containing Kv4.2 and Kv4.3, respectively, in different configurations with KChIP1 fragments lacking the 11-amino-acid aromatic cluster, suggest pore radii at the upper (isoleucine) gate between 4.7 and 7.7 Å, wide enough to let a hydrated K^+^ ion pass. Thus, with the possible exceptions of the “intermediate” and “inactivated” structures put forward by Ye and co-workers, defining an upper (isoleucine) gate for Kv4.2^14^, putative P/C-type inactivated Kv4 channels have not been captured in 3D, yet.

From a physiological point of view, the co-assembly of different Kv4.x channel subtypes with DPPs and a variety of KChIPs, including functionally distinct β-subunit splice variants like 1a and 1b, is expected to contribute to an immense diversity of *I*_SA_ properties in different cell types, with a considerable impact on neuronal excitability and discharge behavior^64^. In the rat brain Kv4.2 is co-localized majorly with KChIP2 and KChIP4 in pyramidal neurons, whereas a high co-localization of Kv4.3 with KChIP1 is seen in large multipolar interneurons^65^. In fact, co-localization of Kv4.3 and KChIP1 is reliably found in a fraction of parvalbumin, calbindin, calretinin and somatostatin-positive hippocampal interneurons, such that Kv4.3/KChIP1 co-expression has been suggested to be used as a separate independent hippocampal interneuron marker^66^. Using siRNA knockdown of KChIP1 expression in a hippocampal preparation has been shown to specifically affect firing behavior of Kv4.3/KChIP1 co-expressing CA1 interneurons. KChIP1 knockdown in these interneurons caused an increase in firing frequency, reportedly due to a slowing of *I*_SA_ recovery rather than a decrease in *I*_SA_ amplitude^47^. Alternative splicing of KChIP1 with the effect of slowing *I*_SA_ recovery may have a comparable effect. The distribution of KChIP1 transcripts, has been studied in human, rat and mouse tissues^26–28^. The findings suggest that the KChIP splice variants 1a and 1b are expressed at comparable amounts in the human brain. It should be noted, that the optional expression of the Kv4.3 splice variants S and L in combination with the KChIP splice variant 1a or 1b may allow for a fairly large spectrum of *I*_SA_ properties in Kv4.3/KChIP1 co-expressing interneurons (see panels B and C in Figs. S4–S6). In the absence of any experimental evidence for the parallel expression of both KChIP1 splice variants in individual cells, 1a + 1b co-expression data suggest the possibility of a graded gating phenotype, producing intermediate *I*_SA_ kinetics (see Table S2).

Taken together, strong promotion of *Shaker*-like P/C-type inactivation features in Kv4 channels, especially by the KChIP splice variant 1b, may limit time-dependent *I*_SA_ availability during repetitive firing, thereby increasing firing frequency, especially in large multipolar Kv4.3/KChIP1 co-expressing interneurons.

## Methods

### Plasmids and constructs

In this study the human Kv4 channel clones Kv4.1, Kv4.2, and Kv4.3^67^ were used. The long Kv4.3 splice variant (Kv4.3 L) was a kind gift from Geoffrey Abbott (Department of Physiology and Biophysics, University of California, Irvine, USA). The human KChIP1a splice variant (referred to as 1a in the present paper) was kindly provided by Dirk Isbrandt (Center for Molecular Medicine, University of Cologne, Germany), and the human DPP6s splice variant (referred to as DPP in the present paper) by Nicole Schmitt (Department of Biomedical Sciences, Faculty of Health and Medical Sciences, Copenhagen, Denmark). In addition to Kv4.2 wild type, three previously studied Kv4.2 mutant constructs were used: In one construct the first ten amino acids had been deleted (Kv4.2 Δ10)^19^; in the two other constructs individual residues in the distal S6 segment, leucine at position 400 or asparagine at position 408, had been replaced by alanine (Kv4.2 L400A and Kv4.2 N408A, respectively^13^; see also Fig. S1). All cDNA clones were inserted into the multiple cloning site of pGEM-HE. In order to generate KChIP1b (referred to as 1b in the present paper), a 33 bp fragment was inserted into the 1a coding region by overlap PCR with appropriate primers (fwd: accagtatcagagagaTAAGATTGAAGATGAGCTGGAG; rev: aataccaccaggcgatgTCTTTCGAGGGTCGCCTT) in a back-to-back orientation, using the Q5 Site Directed Mutagenesis Kit (New England Biolabs). Successful mutagenesis was verified by Sanger sequencing of the complete coding region and flanking sequences. Transformed JM109 *Eschericia coli* cells (Promega) were grown in Luria Broth medium complemented with ampicillin, and plasmids were isolated using the QIAprep Spin Miniprep Kit (QIAGEN). Purified plasmids were linerarized using *Not*I (New England Biolabs), and the RiboMaxLargeScale RNA production system T7 (Promega) was utilized for the in vitro transcription of cRNA.

### Heterologous channel expression

Kv4 channels and their auxiliary β-subunits were expressed in *Xenopus laevis* oocytes. Female frogs (Nasco) were anesthetized for 8–10 min in ethyl 3-aminobenzoate methanesulfonate (tricaine, Sigma; 1.2 g/l chlorine-free frog water containing 7.5 mM Tris-HCl). Part of the ovary lobes was surgically removed, and the wound was immediately sutured (monocryl 4 − 0, ethicon). For the final (sixth, with intervals of ∼ one year) oocyte harvest, the frogs are euthanized by deep tricaine anesthesia (30 min) followed by decapitation. Animal care and experimental procedures related to the harvesting of *Xenopus* oocytes were conducted in accordance with the German Animal Welfare Act and were approved by the Authority for Justice and Consumer Protection of the City of Hamburg (approval # N 101/2023). All procedures comply with the ARRIVE guidelines. The obtained ovary tissue was mechanically dispersed using a pair of fine forceps and digested for 3–5 h under constant agitation in a calcium-free solution containing (in mM) 82.5 NaCl, 2 KCl, 1 MgCl_2_, 5 HEPES, and 1.3 mg/ml collagenase type II (Sigma); pH 7.5, NaOH. Defolliculated stage V–VI oocytes were selected one day after harvesting or later, and 25 or 50 nl cRNA solution were injected per oocyte using a Nanoliter 2000 microinjector (World Precision Instruments). Individual injections resulted in Kv4 cRNA amounts between 0.8 and 5 ng per oocyte, in the absence or presence of KChIP1 cRNA (between 2.5 and 20 ng per oocyte) and/or DPP cRNA (between 2.5 and 5 ng per oocyte). Injected oocytes were incubated at 16 °C in a solution containing (in mM) 75 NaCl, 5 Na-pyruvate, 2 KCl, 2 CaCl_2_, 1 MgCl_2_, 5 HEPES, and 50 mg/ml gentamicin (Sigma), pH 7.5, NaOH; and used for recordings 1–10 days (d1–d10) after cRNA injection (see Tables S1–S4 for cRNA amounts per oocyte and days of recording for individual experiments).

### Electrophysiology

Currents were recorded at room temperature (20–22 °C) under two electrode voltage-clamp, using a TurboTec-3 amplifier (npi electronics) controlled by PatchMaster software (HEKA). The ND96 bath solution contained (in mM) 96 NaCl, 2 KCl, 1 CaCl_2_, 1 MgCl_2_ and 5 HEPES (pH 7.4, NaOH). In some experiments variations of this solution were used, in which either NaCl was replaced by KCl (high K^+^ solution, 98 mM K^+^) or NaCl and KCl were replaced by tetraethylammonium-Cl (TEA solution, 98 mM TEA). Glas microelectrodes were filled with 3 M KCl and had tip resistances of 0.2–0.5 MΩ in standard ND96 bath solution. The holding voltage was − 80 mV. For the study of macroscopic inactivation a 2.5 s test pulse to + 40 mV was applied following a 2 s conditioning pulse to − 100 mV, in order to activate and immediately inactivate a large fraction of channels (see Fig. 1A, B). Recovery from inactivation was measured at − 80 mV using a double-pulse protocol with a 3 s control pulse and a brief test pulse to + 40 mV, separated by interpulse intervals (Δ*t*), which lasted between 10 ms and ∼ 41 s (iteration factor 2, see Fig. 2A, B). Voltage step protocols were applied to study the voltage dependence of gating. For the voltage dependence of activation, test pulses to voltages between − 90 and + 70 mV (10 mV increments, see Fig. 3A, inset) were applied following a 2 s conditioning pulse to -100 mV. For the voltage dependence of steady-state inactivation a double-pulse protocol was used, in which after an initial 2 s conditioning pulse to − 120 mV and a subsequent brief control pulse to + 40 mV, brief test pulses to + 40 mV were applied following an interpulse interval of 10 s with conditioning voltages between − 120 and 0 mV (10 mV increment, see Fig. 3A, inset). Capacitive current transients were not compensated. Current measurements at -95 mV (approximate E_rev_ for K^+^ currents in standard ND96 bath solution), before each voltage pulse protocol, were used to calculate the leak current at any other voltage in order to correct peak current amplitudes. Alternatively, a prepulse-inactivation subtraction protocol was used^38^ to leak-subtract entire current traces.

### Data analysis

The data were analysed using FitMaster (HEKA) and Kaleidagraph (Synergy Software). The kinetics of macroscopic inactivation (i.e., current decay) at + 40 mV and the kinetics of recovery from inactivation (I_test_ / I_control_ plotted against interpulse duration) were described by exponential functions. The sum of three exponential functions was used for macroscopic inactivation kinetics^38,39^ if the mean squared deviation (residual) was decreased to at least 75% in comparison to a double-exponential fit. The sum of two exponential functions was used for recovery kinetics if χ^2^ was decreased to at least 50% in comparison to a single-exponential fit. If these goodness-of-fit criteria were fulfilled, no lower limits were applied to the relative amplitude of individual kinetic components as long as the results were reproducible and the obtained time constants in the milliseconds - seconds range. The relative amplitudes of the individual time constants, obtained with multi-exponential fitting, are given in %. The voltage dependences of activation (cord conductance calculation with E_rev_ = − 95 mV) and steady-state inactivation (I_test_ / I_control_) were analysed with appropriate Boltzmann-functions, as described previously^13^. Fit results (i.e., time constants, relative amplitudes, V_1/2_ values and slope factors) are given as mean ± SD (Figs. 1C and D, 2E and F, 3B and D, 4A and B and 5A and B; see also Tables S1− S5). Normalized current amplitudes in pooled analysis plots containing the fitting curves are given as mean ± SEM (Figs. 2C and D, 3A and C, 4B, D and F and 5B, D and F). Statistical analyses for two groups were performed based on unpaired (comparison of 1a and 1b effects) or paired (effects of solution change while recording from individual oocytes) Student’s *t*-tests. For multiple groups (1a and 1b co-expression effects relative to Kv4.x alone or relative to Kv4.x + DPP) one-way analysis of variance (ANOVA) with Dunnett’s post hoc testing was used (see also Tables S1–S5).

## Supplementary Information

Below is the link to the electronic supplementary material.

Supplementary Material 1

## Data Availability

The data are summarized in Tables S1–S5; data sets generated and analyzed during the present study are available from the corresponding author on reasonable request.
