# Supplementary material for: KChIP1 splice variants modulate Kv4 channels by promoting P/C-type inactivation features
Source: Sci Rep. 2026 Jan 16;16:2632. doi: 10.1038/s41598-026-35770-5 (PMC12823592; doi:10.1038/s41598-026-35770-5)
Supplement: Supplementary file 1 — Supplementary Material 1 [file 41598_2026_35770_MOESM1_ESM.pdf]

KChIP1 splice variants modulate Kv4 channels by promoting P/C-type inactivation features

Wuyou Cao, Georgios Tachtsidis & Robert Bähring

Supplementary information

# - Figures S1 - S7

# - Tables S1 - S5

Cao et al., Fig. S1

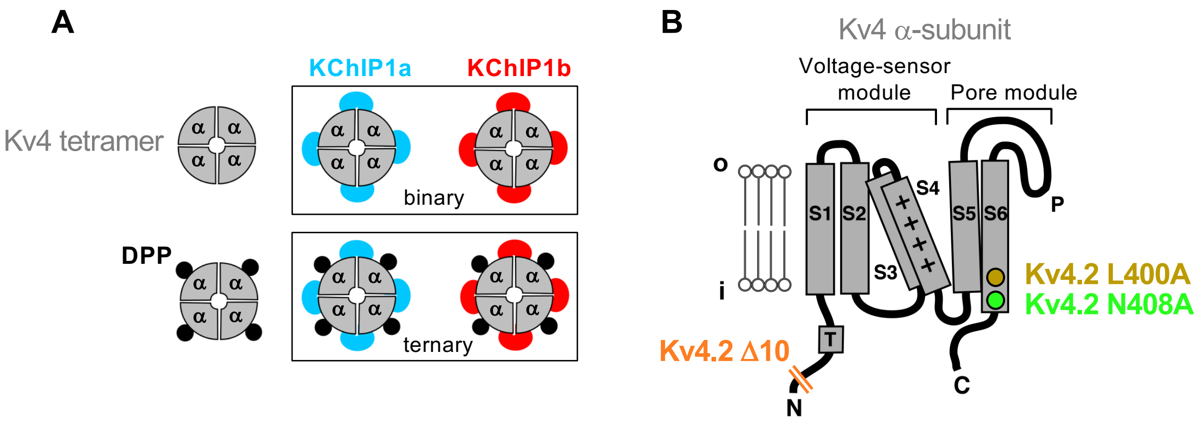

Fig. S1 Kv4 channel subunit composition and structure. Cartoons depict subunit assembly of Kv4 channels and the molecular structure of a Kv4 α-subunit. **A** Tetrameric assembly of Kv4 α-subunits (grey quadrants) in the absence (upper row) and presence of DPP (black circles, lower row); KChIP1 splice variants 1a (blue) or 1b (red) may co-assemble with Kv4 channels in a binary configuration (upper box) or with Kv4 + DPP channels in a ternary configuration (lower box). **B** Membrane topology (lipid bilayer indicated; i: inside; o: outside) and molecular structure of a Kv4 α-subunit with six transmembrane segments (S1 – S6), intracellular N- and C-termini, and an N-terminal tetramerization (T) domain. The α-subunit can be divided into a voltage sensor module (S1 – S4, positively charged amino acid residues in S4) and a pore module (S5 and S6) with a re-entrant pore (P) loop harboring the selectivity filter sequence. In addition to Kv4.2 wild type, a ten amino acid N-terminal deletion construct (Kv4.2 Δ10; Bähring et al., 2001) and two constructs with amino acid substitutions in S6 (Kv4.2 L400A and Kv4.2 N408A; Barghaan & Bähring, 2009) were used in the present study.

References

Bähring, R. et al. Conserved Kv4 N-terminal domain critical for effects of Kv channel-interacting protein 2.2 on channel expression and gating. *J Biol Chem* **276,** 23888-23894 (2001).

Barghaan, J. & Bähring, R. Dynamic coupling of voltage sensor and gate involved in closed-state inactivation of Kv4.2 channels. *J Gen Physiol* **133,** 205-224 (2009).

Cao et al., Fig. S2

```
KChIP1a      -----------------------------------MGA-----VMGTFSSLQT-------   13
KChIP1b      -----------------------------------MGA-----VMGTFSSLQT-------   13
KChIP2a      MRGQGRKESLSDSRDLDGSYDQLTGHPPGPTKKALKQR-----FLKLLPCCGPQALPSVS   55
KChIP3a      MQPAKE-----VTKASDGSLLGDLGHTPLSKKEGIKWQRPRLSRQALMRCCLVKWILS--   53
KChIP4a      -MNVRRVESISAQLEEASSTGG-FLYAQNSTKRSIKER-----LMKLLPCSAAK------   47
                                                            : .

                                                        1  2 3
KChIP1a      ------------KQRRPSKD-----------KIEDELEMTMVCHRPEGLEQLEAQTNFTK   50
KChIP1b      ------------KQRRPSKDIAWWYYQYQRDKIEDELEMTMVCHRPEGLEQLEAQTNFTK   61
KChIP2a      ETLAAPASLRPHRPRLLDPD-----------SVDDEFELSTVCHRPEGLEQLQEQTKFTR  104
KChIP3a      ------------STAPQGSD-----------SSDSELELSTVRHQPEGLDQLQAQTKFTK   90
KChIP4a      ------------TSSPAIQN-----------SVEDELEMATVRHRPEALELLEAQSKFTK   84
                                :           . :.*:*:: * *:**.*: *: *::**:

                            EF1                                 EF2
KChIP1a      RELQVLYRGFKNECPSGVVNEDTFKQIYAQFFPHGDASTYAHYLFNAFDTTQTGSVKFED  110
KChIP1b      RELQVLYRGFKNECPSGVVNEDTFKQIYAQFFPHGDASTYAHYLFNAFDTTQTGSVKFED  121
KChIP2a      KELQVLYRGFKNECPSGIVNEENFKQIYSQFFPQGDSSTYATFLFNAFDTNHDGSVSFED  175
KChIP3a      KELQSLYRGFKNECPTGLVDEDTFKLIYAQFFPQGDATTYAHFLFNAFDADGNGAIHFED  161
KChIP4a      KELQILYRGFKNECPSGVVNEETFKEIYSQFFPQGDSTTYAHFLFNAFDTDHNGAVSFED  155
             :*** **********:*:*:*:.** **:****:**::*** :******:   *:: ***

                                          EF3
KChIP1a      FVTALSILLRGTVHEKLRWTFNLYDINKDGYINKEEMMDIVKAIYDMMGKYTYPVLKEDT  170
KChIP1b      FVTALSILLRGTVHEKLRWTFNLYDINKDGYINKEEMMDIVKAIYDMMGKYTYPVLKEDT  181
KChIP2a      FVAGLSVILRGTVDDRLNWAFNLYDLNKDGCITKEEMLDIMKSIYDMMGKYTYPALREEA  235
KChIP3a      FVVGLSILLRGTVHEKLKWAFNLYDINKDGYITKEEMLAIMKSIYDMMGRHTYPILREDA  221
KChIP4a      FIKGLSILLRGTVQEKLNWAFNLYDINKDGYITKEEMLDIMKAIYDMMGKCTYPVLKEDA  215
             *: .**::*****.::*.*:*****:**** *.****: *:*:******: *** *:*::

                            EF4
KChIP1a      PRQHVDVFFQKMDKNKDGIVTLDEFLESCQEDDNIMRSLQLFQNVM               216
KChIP1b      PRQHVDVFFQKMDKNKDGIVTLDEFLESCQEDDNIMRSLQLFQNVM               227
KChIP2a      PREHVESFFQKMDRNKDGVVTIEEFIESCQKDENIMRSMQLFDNVI               270
KChIP3a      PAEHVERFFEKMDRNQDGVVTIEEFLEACQKDENIMSSMQLFENVI               256
KChIP4a      PRQHVETFFQKMDKNKDGVVTIDEFIESCQKDENIMRSMQLFENVI               250
             * :**: **:***:*:**:**::**:*:**:*:*** *:***:**:
```

Fig. S2 KChIP amino acid sequences. Alignment of KChIP1 (Q9NZI2; 1a and 1b splice variant shown), KChIP2a (Q9NS61), KChIP3a (Q9Y2W7) and KChIP4a (Q6PIL6) amino acid sequences, created with UniProt (https://www.uniprot.org/); numbers on the right indicate amino acid positions. Residues that are perfectly conserved (*) or exhibit either strong (:) or weak (.) similarity are indicated. Both KChIP1a and KChIP1b possess an N-terminal myristoylation motif (green). Note the otherwise highly variable KChIP N-termini but conserved core regions with four EF hand motifs (shaded grey). The 11-amino-acid N-terminal aromatic cluster found in KChIP1b (red), but not in KChIP1a (blue), is captured by none of the available KChIP1 3D structures, starting with His45 (1; Zhou et al., 2004), Glu48 (2; Kise et al., 2021), or Gly49 (3; Ma et al., 2022; Pioletti et al., 2006; Scannevin et al., 2004; Wang et al., 2007).

References

Kise, Y. et al. Structural basis of gating modulation of Kv4 channel complexes. *Nature* **599,** 158-164 (2021).

Ma, D. et al. Structural basis for the gating modulation of Kv4.3 by auxiliary subunits. *Cell Res* **32,** 411-414 (2022).

Pioletti, M., Findeisen, F., Hura, G.L., Minor, D.L. Three-dimensional structure of the KChIP1-Kv4.3 T1 complex reveals a cross-shaped octamer. *Nat Struct Mol Biol* **13,** 987-995 (2006).

Scannevin, R.H. et al. Two N-terminal domains of Kv4 K^+ channels regulate binding to and modulation by KChIP1. *Neuron* **41,** 587-598 (2004).

Wang, H. et al. Structural basis for modulation of Kv4 K^+ channels by auxiliary KChIP subunits. *Nat Neurosci* **10,** 32-39 (2007).

Zhou, W., Qian, Y., Kunjilwar, K., Pfaffinger, P.J., Choe, S. Structural insights into the functional interaction of KChIP1 with *Shal*-type K^+ channels. *Neuron* **41,** 573-586 (2004).

Cao et al., Fig. S3

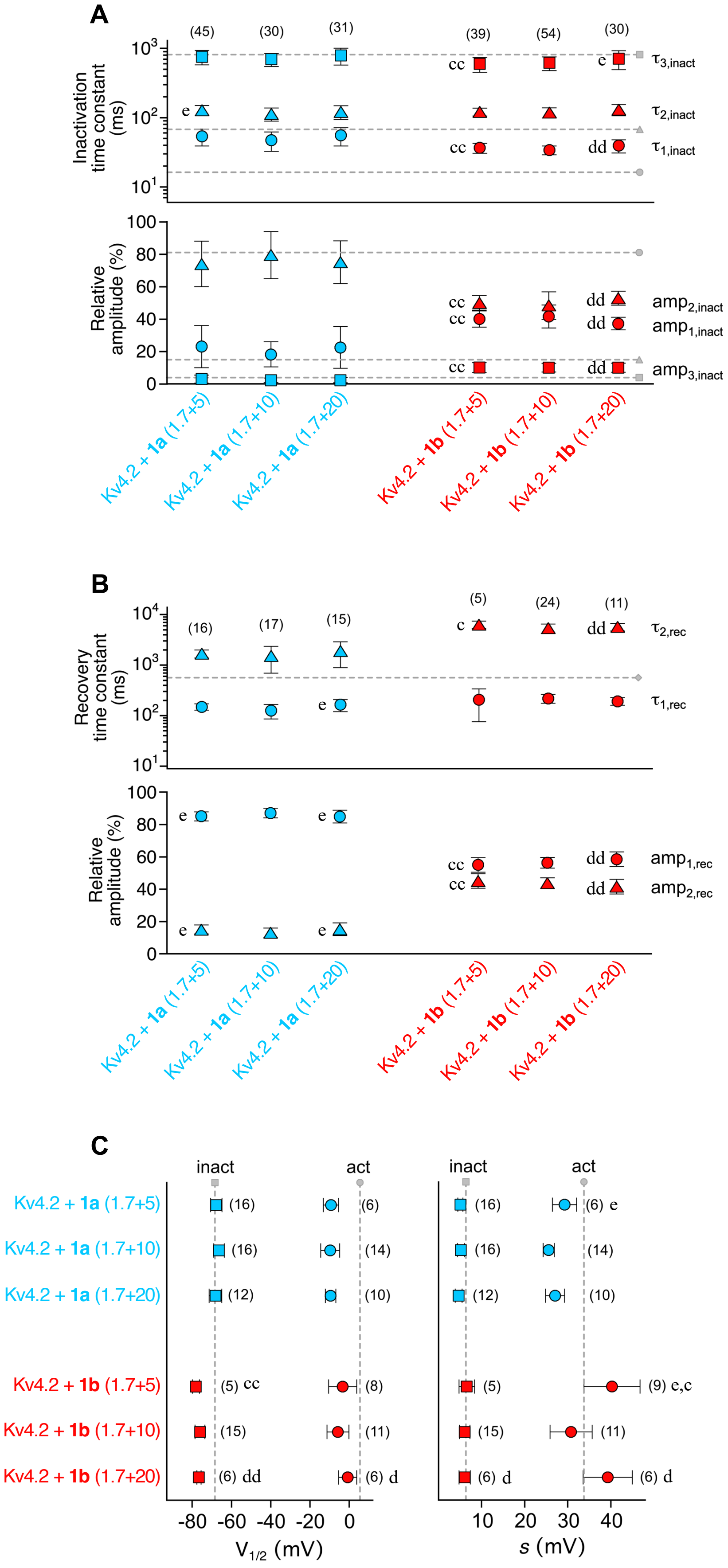

Fig. S3 Extended examination of Kv4.2 channel gating modulation by KChIP1 splice variants. The kinetics of macroscopic inactivation (**A**) and recovery from inactivation (**B**), and the voltage dependences of activation and steady-state inactivation (**C**) were tested for Kv4.2 co-expressed with the KChIP splice variants 1a and 1b at the indicated cRNA amounts (in ng per oocyte). Mean values ± SD and number of oocytes (n) are given for the time constants of macroscopic inactivation and recovery from inactivation (including relative amplitudes in %), and the voltages of halfmaximal activation and inactivation (including slope factors, *s*). Grey dashed lines with symbols indicate the mean values obtained for Kv4.2 alone. Statistical analyses were done using one-way analysis of variance (ANOVA) with Dunnett's post hoc testing for more than two groups and an unpaired Student's *t*-test for two groups. The letters c, d and e denote significant differences; single letter: $p<0.05$; two letters: $p<0.0001$; c,cc: significantly different from Kv4.2 co-expressed with 5 ng 1a cRNA; d, dd: significantly different from Kv4.2 co-expressed with 20 ng 1a cRNA; e: significantly different from Kv4.2 co-expressed with 10 ng KChIP1 cRNA (see also Table S2).

Cao et al., Fig. S4

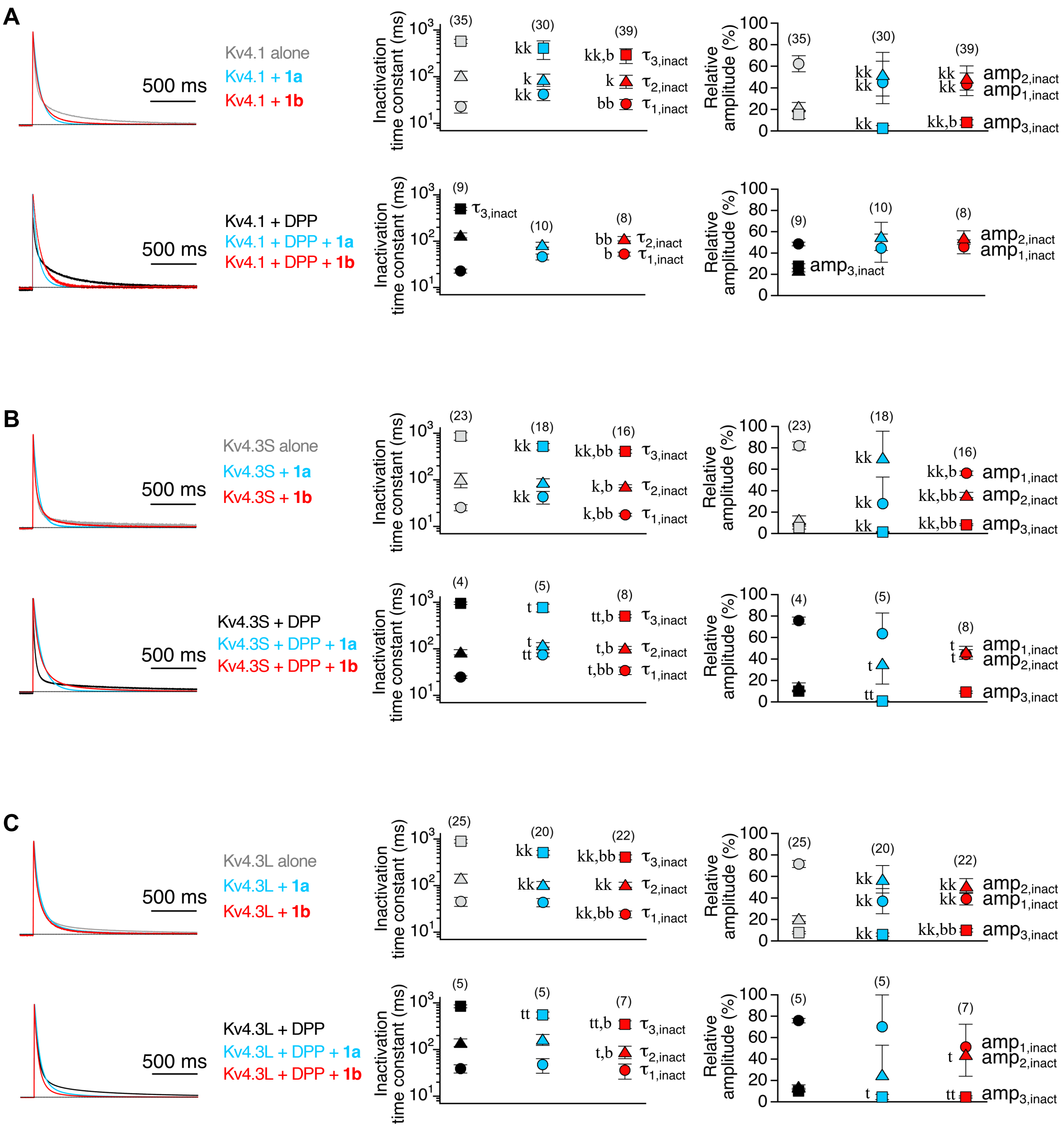

Fig. S4 Modulation of Kv4 channel macroscopic inactivation by KChIP1 splice variants. Kv4 channel macroscopic inactivation kinetics were studied using extended voltage pulses to +40 mV, and the time course of current decay was described by the sum of three exponential functions, except for ternary Kv4.1 channels (two exponentials). Representative current traces and summary graphs are shown for Kv4.1 (**A**), Kv4.3S (**B**) and Kv4.3L (**C**). Currents were normalized to peak and superimposed to demonstrate the effects of KChIP1 splice variant co-expression on current decay kinetics in a binary (upper row) or a ternary (lower row) configuration; Kv4.x alone: grey traces; Kv4.x + DPP: black traces; co-expression with 1a: blue traces; co-expression with 1b: red traces. Inactivation time constants and their relative amplitudes ($\tau_{1,inact}$ and $amp_{1,inact}$: circles; $\tau_{2,inact}$ and $amp_{2,inact}$: triangles; $\tau_{3,inact}$ and $amp_{3,inact}$: squares) are presented as means ± SD; number of oocytes (n) indicated. Significant differences seen with KChIP1 co-expression compared to Kv4.x alone are indicated with k ($p<0.05$) or kk ($p<0.0001$; KChIP1 effects binary; ANOVA); significant differences seen with KChIP1 co-expression compared to Kv4.x + DPP are indicated with t ($p<0.05$) or tt ($p<0.0001$; KChIP1 effects ternary; ANOVA). Significant differences seen with 1b co-expression (compared to 1a co-expression; i.e., special features of the 1b splice variant) are indicated with b ($p<0.05$) or bb ($p<0.0001$; unpaired Student's *t*-test; see also Tables S1, S3 and S4).

Cao et al., Fig. S5

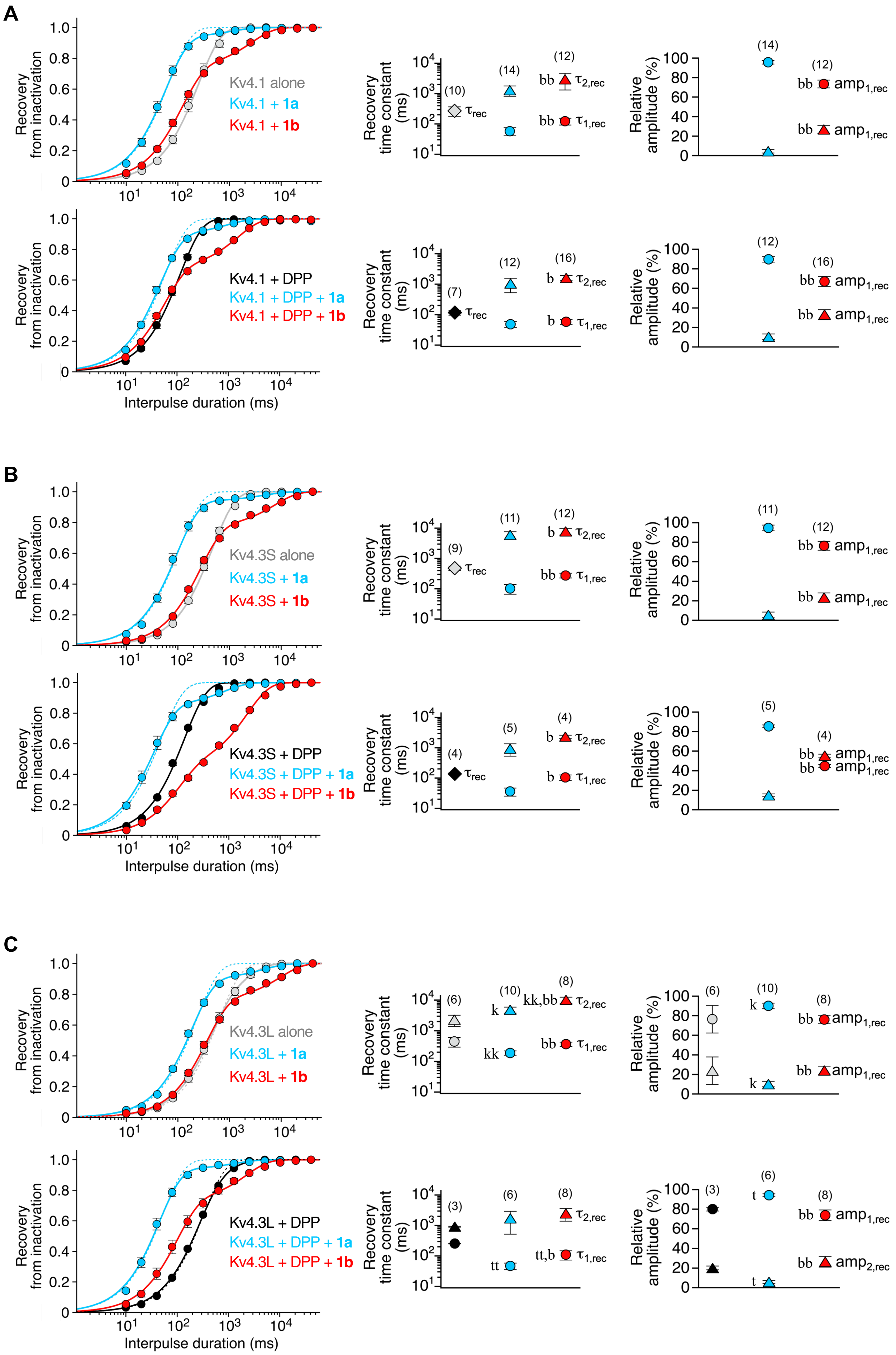

Fig. S5 Modulation of Kv4 channel recovery kinetics by KChIP1 splice variants. Recovery of Kv4 channels from inactivation was measured at -80 mV. Recovery plots and summary graphs are shown for Kv4.1 (**A**), Kv4.3S (**B**) and Kv4.3L (**C**). Relative current amplitudes (I_{test} / $I_{control}$; error bars are SEM) were plotted against interpulse duration, and the data were described by exponential functions: A single-exponential function was sufficient for the recovery kinetics of Kv4.x alone (grey) and Kv4.x + DPP (black), except for Kv4.3L, which required a double-exponential function in the absence of KChIP1. A double-exponential function was also necessary for the recovery kinetics of Kv4.x + KChIP1 and Kv4.x + DPP + KChIP1 (1a: blue; 1b: red); Dotted lines: single-exponential functions fitted to biphasic recovery data. Recovery time constants from single-exponential fits (τ_{rec}, diamonds) and time constants including their relative amplitudes from double-exponential fits ($\tau_{1,rec}$ and $amp_{1,rec}$: circles; $\tau_{2,rec}$ and $amp_{2,rec}$: triangles) are presented as means $\pm$ SD; number of oocytes (n) indicated. Significant differences seen with KChIP1 co-expression compared to Kv4.x alone are indicated with k ($p<0.05$) or kk ($p<0.0001$; KChIP1 effects binary); significant differences seen with KChIP1 co-expression compared to Kv4.x + DPP are indicated with t ($p<0.05$) or tt ($p<0.0001$; KChIP1 effects ternary; ANOVA). Significant differences seen with 1b co-expression (compared to 1a co-expression; i.e., special features of the 1b splice variant) are indicated with b ($p<0.05$) or bb ($p<0.0001$; unpaired Student's *t*-test; see also Tables S1, S3 and S4).

Cao et al., Fig. S6

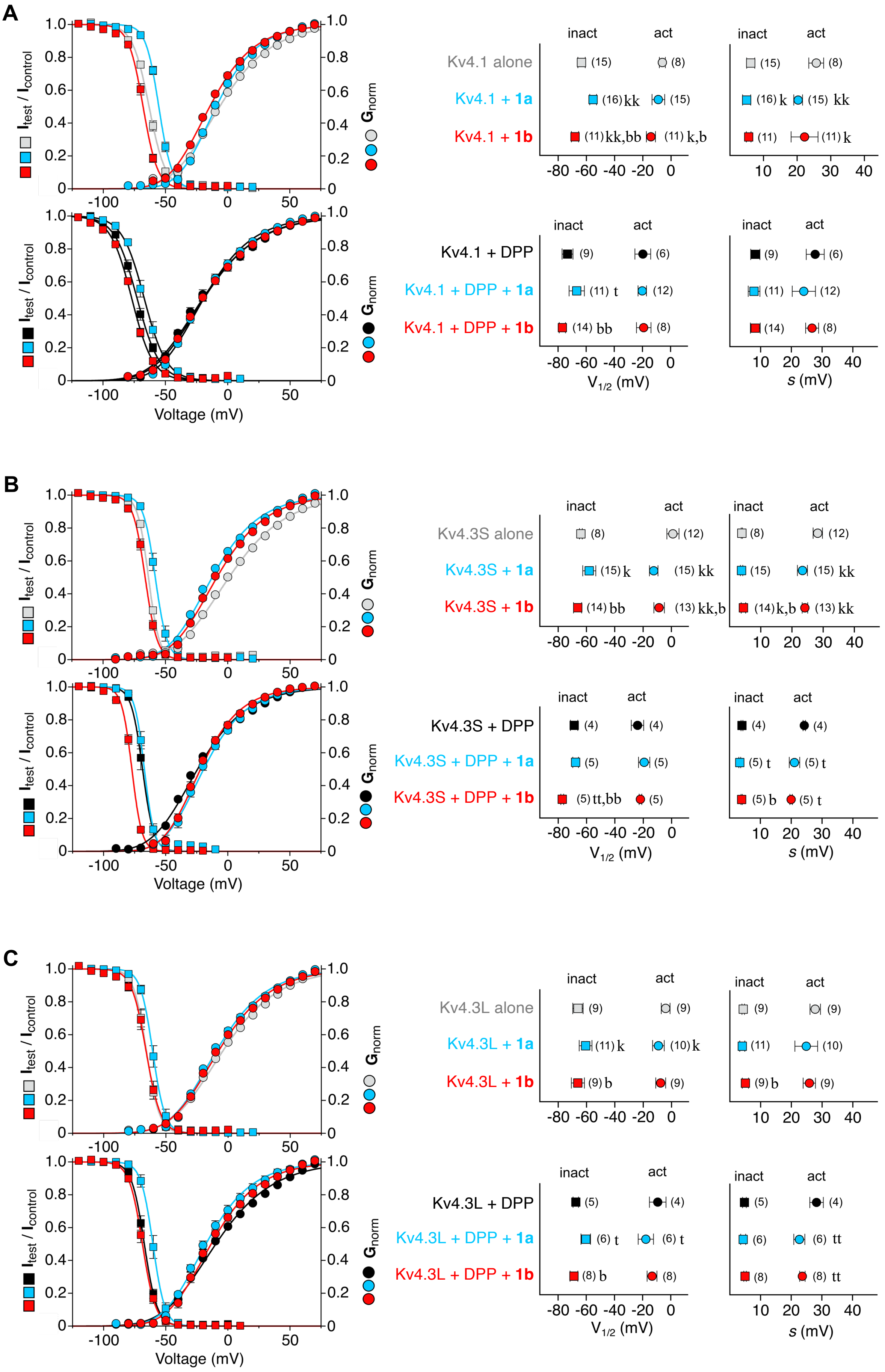

Fig. S6 Effects of KChIP1 splice variants on the voltage dependence of Kv4 channel gating. The voltage dependences of activation and steady-state inactivation were measured. Activation and inactivation curves, and summary graphs are shown for Kv4.1 (**A**), Kv4.3S (**B**) and Kv4.3L (**C**). Normalized data (I_{test} / $I_{control}$) from steady-state inactivation protocols and normalized conductance values (G_{norm}) from activation protocols were plotted against conditioning or test pulse voltage, respectively, in the same graph (error bars are SEM); upper graphs: Kv4.x alone (grey) and binary channels with 1a (blue) or 1b (red); lower graphs: Kv4.2 + DPP (black) and ternary channels with 1a (blue) or 1b (red). Voltages of halfmaximal activation (act, circles) and inactivation (inact, squares) and corresponding slope factors ($V_{1/2}$ and s, respectively) obtained with Boltzmann-analysis are presented as means ± SD; numbers of oocytes (n) indicated. Significant differences seen with KChIP1 co-expression compared to Kv4.x alone are indicated with k ($p<0.05$) or kk ($p<0.0001$; KChIP1 effects binary); significant differences seen with KChIP1 co-expression compared to Kv4.x + DPP are indicated with t ($p<0.05$) or tt ($p<0.0001$; KChIP1 effects ternary; ANOVA). Significant differences seen with 1b co-expression (compared to 1a co-expression; i.e., special features of the 1b splice variant) are indicated with b ($p<0.05$) or bb ($p<0.0001$; unpaired Student's *t*-test; see also Tables S1, S3 and S4).

Cao et al., Fig. S7

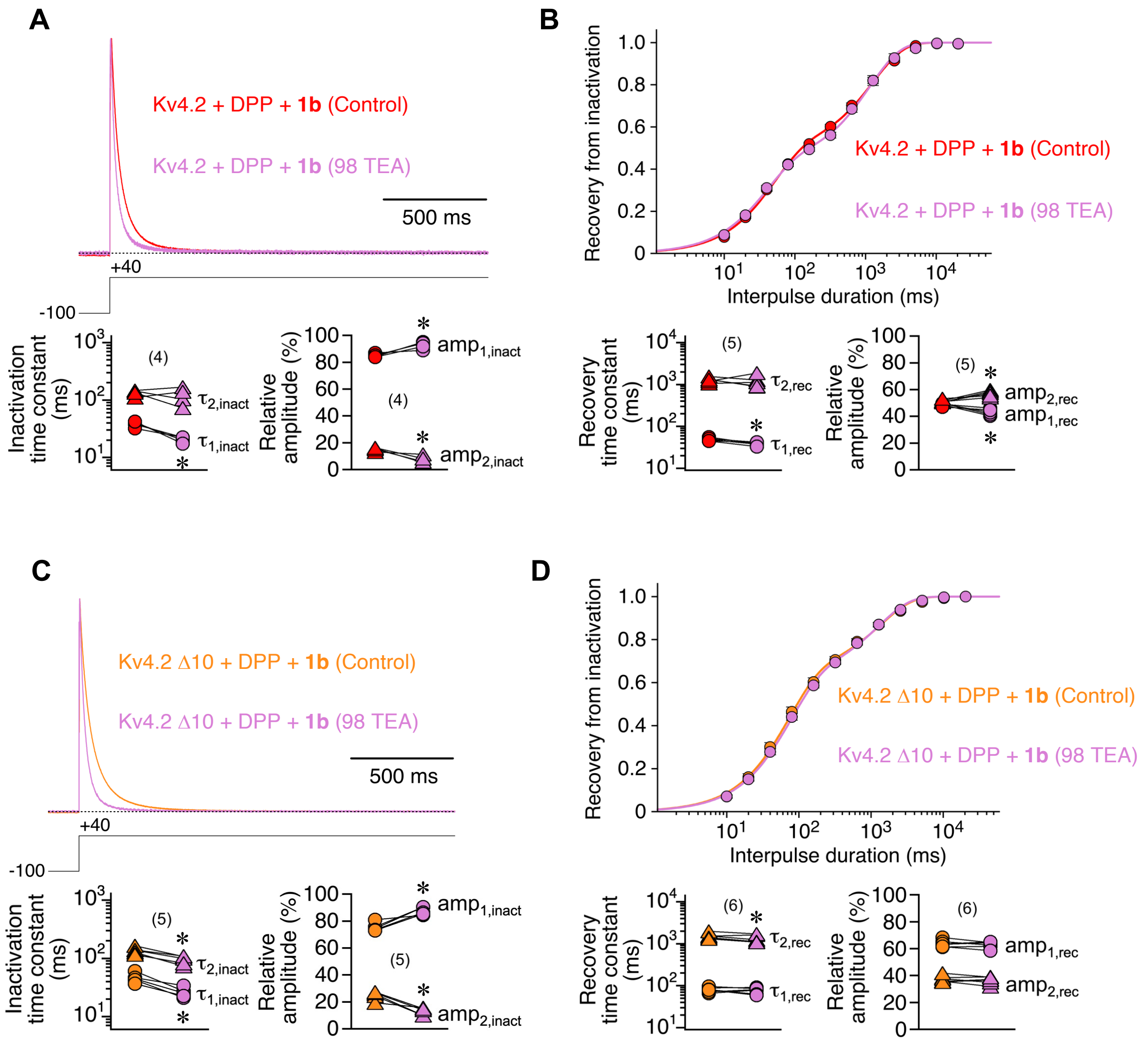

Fig. S7 Effect of TEA on the macroscopic inactivation and recovery kinetics of wild-type and mutant ternary Kv4.2 + DPP + 1b channels. The kinetics of macroscopic inactivation and recovery from inactivation were analysed for wild-type and N-terminally truncated Kv4.2 channels (deletion of the first ten amino acids, Δ10) co-expressed with DPP and 1b. Recordings were obtained from individual oocytes in standard bath solution (Control) and in the presence of a high concentration (98 mM) of TEA. The time course of current decay and the time course of recovery from inactivation were described by exponential functions. **A** Currents of an oocyte expressing wild-type Kv4.2 + DPP + 1b channels recorded in control solution (red) and TEA (purple) were normalized to peak and superimposed. Macroscopic inactivation kinetics were approximated by a double-exponential function. Corresponding inactivation time constants and their relative amplitudes ($\tau_{1,inact}$ and $amp_{1,inact}$: circles; $\tau_{2,inact}$ and $amp_{2,inact}$: triangles) obtained under the two conditions are shown below (data pairs indicated). **B** Recovery plots for wild-type Kv4.2 + DPP + 1b including double-exponential fits obtained in control solution (red) and TEA (purple). Corresponding recovery time constants and their relative amplitudes ($\tau_{1,rec}$ and $amp_{1,rec}$: circles; $\tau_{2,rec}$ and $amp_{2,rec}$: triangles) obtained under the two conditions are shown below (data pairs indicated). **C** and **D** Same measurements and analysis for Kv4.2 Δ10 + DPP + 1b in control solution (orange) and TEA (purple); All time constants and relative amplitudes are presented as means ± SD; number of oocytes (n) indicated. In the recovery plots the data are presented as means ± SEM. Recordings of the same oocyte in control solution and TEA were statistically analysed using a paired Student's *t*-test. Significant effects of switching from control to TEA are indicated as *($p<0.05$; see also Table S5).

Table S1. Functional characterization of Kv4.1 in the absence and presence of KChIP1 splice variants 1a and 1b in different channel configurations.

<table>
<tr><th>Kv4.1 configuration
(ng cRNA, day)</th><th>$\tau_{1,inact}$ / $\tau_{f,inact}$
(ms)</th><th>$amp_{1,inact}$ / $amp_{f,inact}$
(%)</th><th>$\tau_{2,inact}$ / $\tau_{s,inact}$
(ms)</th><th>$amp_{2,inact}$ / $amp_{s,inact}$
(%)</th><th>$\tau_{3,inact}$
(ms)</th><th>$amp_{3,inact}$
(%)</th><th>$\tau_{1,rec}$ / τ_{rec}
(ms)</th><th>$amp_{1,rec}$
(%)</th><th>$\tau_{2,rec}$
(ms)</th><th>$amp_{2,rec}$
(%)</th><th>$V_{1/2\ act}$
(mV)</th><th>s_{act}
(mV)</th><th>$V_{1/2\ inact}$
(mV)</th><th>s_{inact}
(mV)</th></tr>
<tr><td>Kv4.1 alone
(5, d2-d3)</td><td>22.9 ± 6.4
(35)</td><td>62 ± 8
(35)</td><td>107 ± 25
(35)</td><td>23 ± 4
(35)</td><td>570 ± 57
(35)</td><td>15 ± 4
(35)</td><td>270 ± 93
(10)</td><td colspan="3">-</td><td>-6.1 ± 2.1
(8)</td><td>25.7 ± 2.2
(8)</td><td>-63.4 ± 2.6
(15)</td><td>6.2 ± 1.2
(15)</td></tr>
<tr><td>Kv4.1 + 1a
(1.7+10, d2)</td><td rowspan="2">41.9 ± 11.3^{kk}
(30)</td><td rowspan="2">45 ± 20^{kk}
(30)</td><td rowspan="2">87.4 ± 25.5^{k}
(30)</td><td rowspan="2">53 ± 20^{kk}
(30)</td><td rowspan="2">405 ± 174^{kk}
(30)</td><td rowspan="2">2 ± 2^{kk}
(30)</td><td rowspan="2">57.6 ± 17.3
(14)</td><td rowspan="2">96 ± 2
(14)</td><td rowspan="2">1300 ± 491
(14)</td><td rowspan="2">4 ± 2
(14)</td><td>-9.0 ± 4.5
(15)</td><td>20.3 ± 1.3^{kk}
(15)</td><td>-55.2 ± 2.4^{kk}
(16)</td><td>5.0 ± 1.2^{k}
(16)</td></tr>
<tr><td>Kv4.1 + 1a
(1.7+10, d3)</td><td colspan="4">-</td></tr>
<tr><td>Kv4.1 + 1b
(1.7+10, d2)</td><td rowspan="2">26.0 ± 6.4^{bb}
(39)</td><td rowspan="2">43 ± 11^{kk}
(39)</td><td rowspan="2">81.9 ± 25.6^{k}
(39)</td><td rowspan="2">49 ± 11^{kk}
(39)</td><td rowspan="2">$292 \pm 103^{kk,b}$
(39)</td><td rowspan="2">$8 \pm 23^{kk,b}$
(39)</td><td rowspan="2">123 ± 31.4^{bb}
(12)</td><td rowspan="2">73 ± 4^{bb}
(12)</td><td rowspan="2">2979 ± 1661^{bb}
(12)</td><td rowspan="2">27 ± 4^{bb}
(12)</td><td rowspan="2">$-14.2 \pm 3.2^{k,b}$
(11)</td><td rowspan="2">22.2 ± 4.0^{k}
(11)</td><td rowspan="2">$-68.0 \pm 2.5^{kk,bb}$
(11)</td><td rowspan="2">5.6 ± 0.7
(11)</td></tr>
<tr><td>Kv4.1 + 1b
(1.7+10, d3)</td></tr>
<tr><td>Kv4.1 + DPP
(1.7+5, d1)</td><td>22.7 ± 2.2
(9)</td><td>48 ± 2
(9)</td><td>134 ± 16
(9)</td><td>24 ± 2
(9)</td><td>498 ± 37
(9)</td><td>28 ± 2
(9)</td><td rowspan="2">116 ± 21
(7)</td><td rowspan="2" colspan="3">-</td><td rowspan="2">-19.6 ± 5.6
(6)</td><td rowspan="2">27.8 ± 2.9
(6)</td><td rowspan="2">-73.2 ± 3.9
(9)</td><td rowspan="2">8.3 ± 1.5
(9)</td></tr>
<tr><td>Kv4.1 + DPP
(1.7+5, d2)</td><td colspan="6">-</td></tr>
<tr><td>Kv4.1 + DPP + 1a
(1.7+5+10, d1)</td><td>45.8 ± 6.6
(10)</td><td>46 ± 13
(10)</td><td>84.2 ± 9.9
(10)</td><td>54 ± 13
(10)</td><td colspan="2">-</td><td rowspan="2">48.2 ± 10.3
(12)</td><td rowspan="2">90 ± 3
(12)</td><td rowspan="2">1042 ± 521
(12)</td><td rowspan="2">10 ± 3
(12)</td><td rowspan="2">-20.1 ± 3.0
(12)</td><td rowspan="2">24.1 ± 3.8
(12)</td><td rowspan="2">-66.7 ± 5.5^{t}
(11)</td><td rowspan="2">7.9 ± 1.9
(11)</td></tr>
<tr><td>Kv4.1 + DPP + 1a
(1.7+5+10, d2)</td><td colspan="6">-</td></tr>
<tr><td>Kv4.1 + DPP + 1b
(1.7+5+10, d1)</td><td>52.6 ± 5.1^{b}
(8)</td><td>46 ± 7
(8)</td><td>113 ± 12^{bb}
(8)</td><td>54 ± 7
(8)</td><td colspan="2" rowspan="2">-</td><td rowspan="3">58.0 ± 10.3^{b}
(16)</td><td rowspan="3">67 ± 5^{bb}
(16)</td><td rowspan="3">1605 ± 311^{b}
(16)</td><td rowspan="3">33 ± 5^{bb}
(16)</td><td rowspan="3">-19.0 ± 5.1
(8)</td><td rowspan="3">26.8 ± 2.1
(8)</td><td rowspan="3">-76.9 ± 2.2^{bb}
(14)</td><td rowspan="3">8.3 ± 1.6
(14)</td></tr>
<tr><td>Kv4.1 + DPP + 1b
(1.7+5+10, d2)</td><td colspan="4">-</td></tr>
<tr><td>Kv4.1 + DPP + 1b
(1.7+5+10, d5)</td><td colspan="6">-</td></tr>
</table>

Kv4.1 was expressed alone or in different channel configurations (i.e., in the absence or presence of DPP) with either KChIP1a (1a) or KChIP1b (1b) in *Xenopus* oocytes (cRNA amounts in ng per oocyte indicated). Functional characterization under two-electrode voltage-clamp was performed on day 1 - 5 after cRNA injection (*d1 - d5*, as indicated). Mean values ± SD and number of oocytes (n) are given for the time constants of macroscopic inactivation and recovery from inactivation (including relative amplitudes in %), and the voltages of halfmaximal activation and steady-state inactivation (including slope factors, *s*). Statistical analyses were done using one-way analysis of variance (ANOVA) with Dunnett's post hoc testing for more than two groups and an unpaired Student's *t*-test for two groups. Superscript letters k, b and t denote significant differences; single letter: $p<0.05$; two letters: $p<0.0001$; k, kk: significantly different from Kv4.1 alone (KChIP effect, binary); b, bb: significantly different from 1a co-expression (special feature of the 1b splice variant); t,tt: significantly different from Kv4.1 + DPP (KChIP effect in the presence of DPP, ternary).

Table S2. Functional characterization of Kv4.2 in the absence and presence of KChIP1 splice variants 1a and 1b in different channel configurations.

Kv4.2 configuration (ng cRNA, day)	$\tau_{1,inact}$ (ms)	$amp_{1,inact}$ (%)	$\tau_{2,inact}$ (ms)	$amp_{2,inact}$ (%)	$\tau_{3,inact}$ (ms)	$amp_{3,inact}$ (%)	**$\tau_{1,rec}$ / τ_{rec} (ms)**	**$amp_{1,rec}$ (%)**	**$\tau_{2,rec}$ (ms)**	**$amp_{2,rec}$ (%)**	$V_{1/2\ act}$ (mV)	*s* act (mV)	$V_{1/2\ inact}$ (mV)	*s* inact (mV)
Kv4.2 alone (5, *d2-d10*)	16.2 ± 2.1 (30)	81 ± 3 (30)	67.7 ± 11.4 (30)	15 ± 2 (30)	809 ± 128 (30)	4 ± 2 (30)	**563 ± 76 (16)**	-			5.5 ± 5.0 (12)	33.8 ± 2.5 (12)	-68.3 ± 2.2 (14)	6.4 ± 0.9 (14)
Kv4.2 + **1a** (1.7+5, *d2*)	53.4 ± 14.5^{kk} (45)	23 ± 13^{kk} (45)	128 ± 21^{kk,e} (45)	74 ± 14^{kk} (45)	753 ± 175 (45)	3 ± 3 (45)	**149 ± 22 (16)**	**85 ± 3^{e} (16)**	**1674 ± 296 (16)**	**15 ± 3^{e} (16)**	-9.2 ± 3.9^{kk} (6)	29.3 ± 2.8^{k} (6)	-67.7 ± 2.9 (16)	5.1 ± 0.6^{k} (16)
Kv4.2 + **1a** (1.7+10, *d2*)	47.1 ± 14.6^{kk} (30)	18 ± 8^{kk} (30)	113 ± 24.3^{kk} (30)	80 ± 15^{kk} (30)	697 ± 150 (30)	2 ± 1^{k} (30)	**126 ± 40 (17)**	**87 ± 3 (17)**	**1514 ± 825 (17)**	**13 ± 3 (17)**	-9.6 ± 4.8^{kk} (14)	25.6 ± 1.3^{kk} (14)	-66.3 ± 2.7 (16)	5.2 ± 0.8^{k} (16)
Kv4.2 + **1a** (1.7+20, *d2*)	55.2 ± 16.3^{kk} (31)	23 ± 13^{kk} (31)	121 ± 27^{kk} (31)	75 ± 13^{kk} (31)	789 ± 216 (31)	2 ± 2^{k} (31)	**164 ± 44^{e} (15)**	**85 ± 4^{e} (15)**	**1893 ± 1002 (15)**	**15 ± 4^{e} (15)**	-9.4 ± 2.7^{kk} (10)	27.1 ± 2.2^{kk} (10)	-68.1 ± 3.2 (12)	4.7 ± 0.8^{k} (12)
Kv4.2 + **1b** (1.7+5, *d2*)<br>Kv4.2 + **1b** (1.7+5, *d3*)	36.5 ± 6.1^{kk,cc} (39)	40 ± 5^{kk,cc} (39)	121 ± 16.1^{kk} (39)	50 ± 5^{kk,cc} (39)	594 ± 145^{kk,cc} (39)	10 ± 3^{kk,cc} (39)	**207 ± 131 (5)**	**55 ± 5^{cc} (5)**	**6325 ± 1047^{c} (5)**	**45 ± 5^{cc} (5)**	-3.3 ± 7.2^{kk} (8)	40.3 ± 6.5^{e,c} (8)	-78.1 ± 1.8^{kk,cc} (5)	6.6 ± 1.8 (5)
Kv4.2 + **1b** (1.7+10, *d2*)<br>Kv4.2 + **1b** (1.7+10, *d3*)	33.9 ± 5^{kk,bb} (54)	42 ± 7^{kk,bb} (54)	117.9 ± 20.0^{kk} (54)	48 ± 9^{kk,bb} (54)	615 ± 137^{kk,b} (54)	10 ± 3^{kk,bb} (54)	**218 ± 43^{bb} (24)**	**56 ± 3^{bb} (24)**	**5347 ± 1125^{bb} (24)**	**44 ± 4^{bb} (24)**	-5.7 ± 5.6^{k,b} (11)	30.8 ± 4.9^{b} (11)	-76.0 ± 2.5^{kk,bb} (15)	6.1 ± 1.2^{b} (15)
Kv4.2 + **1b** (1.7+20, *d2*)<br>Kv4.2 + **1b** (1.7+20, *d3*)	39.3 ± 8.5^{kk,dd} (30)	37 ± 4^{kk,dd} (30)	130 ± 24^{kk} (30)	53 ± 4^{kk,dd} (30)	707 ± 214^{k,e} (30)	10 ± 3^{kk,dd} (30)	**192 ± 33 (11)**	**59 ± 5^{dd} (11)**	**5658 ± 945^{dd} (11)**	**41 ± 5^{dd} (11)**	-0.7 ± 4.6^{kk,d} (6)	39.3 ± 5.7^{d} (6)	-76.5 ± 1.1^{kk,dd} (6)	6.1 ± 1.3^{d} (6)
Kv4.2 + DPP (1.7+5, *d1*)	9.1 ± 1.9 (10)	86 ± 6 (10)	50.0 ± 33.8 (10)	11 ± 6 (10)	1136 ± 156 (10)	3 ± 1 (10)	**117 ± 32 (14)**	-			-28.5 ± 5.0 (8)	23.2 ± 5 (8)	-73.5 ± 1.5 (6)	4.7 ± 0.2 (6)
Kv4.2 + DPP (1.7+5, *d2*)	-													
Kv4.2 + DPP + **1a** (1.7+5+10, *d1*)	31.7 ± 3.6^{tt,h} (7)	41 ± 7^{tt,hh} (7)	64.5 ± 3.0 (7)	57 ± 7^{tt,hh} (7)	531 ± 32^{tt} (7)	2 ± 1^{t} (7)	-							
Kv4.2 + DPP + **1a** (1.7+5+10, *d2*)	-						**24.2 ± 6.6 (7)**	**79 ± 4^{hh} (7)**	**395 ± 128^{h} (7)**	**21 ± 4^{hh} (7)**	-19.2 ± 3.6^{t} (6)	26.1 ± 2.1 (6)	-74.4 ± 2.6 (7)	4.3 ± 0.3 (7)
Kv4.2 + DPP + **1b** (1.7+5+10, *d1*)	32.8 ± 3.5^{tt} (3)	48 ± 3^{tt,b} (3)	84.6 ± 5.8^{b} (3)	48 ± 2^{tt,b} (3)	483 ± 98^{tt} (3)	4 ± 1 (3)	-							
Kv4.2 + DPP + **1b** (1.7+5+10, *d2*)	32.0 ± 3.0^{tt,h} (17)	51 ± 3^{tt,b,hh} (17)	77.4 ± 6.4^{t,bb,hh} (17)	45 ± 3^{tt,b,hh} (17)	513 ± 40^{tt,h} (17)	4 ± 1^{bb,hh} (17)	**41.4 ± 4.2^{b,hh} (8)**	**57 ± 4^{bb,hh} (8)**	**1275 ± 167^{bb,hh} (8)**	**43 ± 4^{bb,hh} (8)**	-12.1 ± 3.6^{tt,b} (7)	33.7 ± 2.4^{tt,bb} (7)	-81.3 ± 1.5^{tt,b} (9)	7.4 ± 1.0^{tt,bb} (9)
Kv4.2 + DPP + **1a** + **1b** (1.7+5+5+5, *d1+d2*)	27.8 ± 3.5 (22)	68 ± 13 (22)	63.3 ± 11.4 (22)	30 ± 13 (22)	581 ± 66 (22)	2 ± 1 (22)	**28.7 ± 5.7 (22)**	**69 ± 3 (22)**	**577 ± 135 (22)**	**31 ± 3 (22)**	-			

Kv4.2 was expressed alone or in different channel configurations (i.e., in the absence or presence of DPP) with either KChIP1a (1a) or KChIP1b (1b) in *Xenopus* oocytes (cRNA amounts in ng per oocyte indicated). Functional characterization under two-electrode voltage-clamp was performed on day 1 - 10 after cRNA injection (*d1 - d10*, as indicated). Mean values ± SD and number of oocytes (n) are given for the time constants of macroscopic inactivation and recovery from inactivation (including relative amplitudes in %), and the voltages of halfmaximal activation and inactivation (including slope factors, *s*). Statistical analyses were done using one-way analysis of variance (ANOVA) with Dunnett's post hoc testing for more than two groups and an unpaired Student's *t*-test for two groups. Superscript letters k, b, c, d, e, t and h denote significant differences; single letter: $p<0.05$; two letters: $p<0.0001$; k, kk: significantly different from Kv4.2 alone (KChIP effect, binary); b, bb: significantly different from Kv4.2 co-expressed with 10 ng 1a cRNA in the absence or presence of DPP; c,cc: significantly different from Kv4.2 co-expressed with 5 ng 1a cRNA; d, dd: significantly different from Kv4.2 co-expressed with 20 ng 1a cRNA (b, c and d indicate special features of the 1b splice variant); e: significantly different from Kv4.2 co-expressed with 10 ng KChIP1 cRNA; t,tt: significantly different from Kv4.2 + DPP (KChIP effect in the presence of DPP, ternary); h,hh: significantly different from Kv4.2 + DPP + 1a + 1b (i.e., co-expression of 1a and 1b in a heteromeric ternary configuration).

Table S3. Functional characterization of Kv4.3S in the absence and presence of KChIP1 splice variants 1a and 1b in different channel configurations.

Kv4.3S configuration (ng cRNA, day)	$\tau_{1,inact}$ (ms)	$amp_{1,inact}$ (%)	$\tau_{2,inact}$ (ms)	$amp_{2,inact}$ (%)	$\tau_{3,inact}$ (ms)	$amp_{3,inact}$ (%)	**$\tau_{1,rec}$ / τ_{rec} (ms)**	**$amp_{1,rec}$ (%)**	**$\tau_{2,rec}$ (ms)**	**$amp_{2,rec}$ (%)**	$V_{1/2\ act}$ (mV)	s_{act} (mV)	$V_{1/2\ inact}$ (mV)	s_{inact} (mV)
Kv4.3S alone (0.8, *d2*)	25.3 ± 3.8 (23)	82 ± 4 (23)	103 ± 35.4 (23)	13 ± 4 (23)	858 ± 156 (23)	5 ± 1 (23)	-							
Kv4.3S alone (1.7, *d2*)	-						**478 ± 82.3 (9)**	-			1.2 ± 4.3 (12)	28.3 ± 1.5 (12)	-63.9 ± 1.0 (8)	4.0 ± 0.2 (8)
Kv4.3S + **1a** (0.8+5, d2)	43.1 ± 13.2^{kk} (18)	28 ± 25^{kk} (18)	88.5 ± 16.2 (18)	71 ± 25^{kk} (18)	518 ± 82.4^{kk} (18)	1 ± 1^{kk} (18)	-							
Kv4.3S + **1a** (1.7+10, *d2*)	-						**102 ± 36.4 (11)**	**95 ± 3 (11)**	**5970 ± 1625 (11)**	**5 ± 3 (11)**	-12.4 ± 3.0^{kk} (15)	23.4 ± 1.6^{kk} (15)	-58.0 ± 4.6^{k} (15)	3.9 ± 0.4 (15)
Kv4.3S + **1b** (0.8+5, *d2*)	17.8 ± 1.2^{k,bb} (16)	56 ± 2^{kk,b} (16)	73.1 ± 5.4^{k,b} (16)	36 ± 3^{kk,bb} (16)	408 ± 36.9^{kk,bb} (16)	8 ± 1^{kk,bb} (16)	-							
Kv4.3S + **1b** (1.7+10, *d2*)							**274 ± 47.6^{bb} (12)**	**76 ± 5^{bb} (12)**	**7826 ± 1931^{b} (12)**	**24 ± 5^{bb} (12)**	-8.6 ± 3.7^{kk,b} (13)	24.2 ± 0.9^{kk} (13)	-66.3 ± 2.9^{bb} (14)	4.5 ± 0.7^{k,b} (14)
Kv4.3S + DPP (0.8+2.5, *d2*)	24.6 ± 1.5 (4)	76 ± 3 (4)	84.8 ± 10.8 (4)	14 ± 3 (4)	949 ± 58.9 (4)	10 ± 1 (4)	**136 ± 11.0 (4)**	-			-24.0 ± 4.4 (4)	24.3 ± 0.4 (4)	-68.7 ± 2.6 (4)	4.1 ± 0.3 (4)
Kv4.3S + DPP + **1a** (1.7+5+10, *d1*)	74.3 ± 7.1^{tt} (5)	63 ± 19 (5)	122 ± 13.4^{t} (5)	36 ± 19^{t} (5)	764 ± 148^{t} (5)	1 ± 0.2^{tt} (5)	-							
Kv4.3S + DPP + **1a** (0.8+2.5+5, *d2*)	-						**35.7 ± 10.4 (5)**	**85 ± 2 (5)**	**930 ± 408 (5)**	**15 ± 2 (5)**	-19.3 ± 4.0 (5)	21.1 ± 1.7^{t} (5)	-67.7 ± 2.0 (5)	3.4 ± 0.3^{t} (5)
Kv4.3S + DPP + **1b** (1.7+5+10, *d2*)	34.1 ± 6.1^{t,bb} (8)	44 ± 4^{t} (8)	105 ± 8.1^{t,b} (8)	47 ± 5^{t} (8)	501 ± 35.8^{tt,b} (8)	9 ± 1 (8)	-							
Kv4.3S + DPP + **1b** (0.8+2.5+5, *d2*)	-						**104 ± 21.9^{b} (4)**	**45 ± 2^{bb} (4)**	**2309 ± 277^{b} (4)**	**55 ± 2^{bb} (4)**	-21.9 ± 1.0 (5)	20.0 ± 0.4^{t} (5)	-77.1 ± 1.0^{tt,bb} (5)	4.0 ± 0.2^{b} (5)

Kv4.3S (short splice variant) was expressed alone or in different channel configurations (i.e., in the absence or presence of DPP) with either KChIP1a (1a) or KChIP1b (1b) in *Xenopus* oocytes (cRNA amounts in ng per oocyte indicated). Functional characterization under two-electrode voltage-clamp was performed on day 1 - 2 after cRNA injection (*d1* - *d2*, as indicated). Mean values ± SD and number oocytes (n) are given for the time constants of macroscopic inactivation and recovery from inactivation (including relative amplitudes in %), and the voltages of halfmaximal activation and inactivation (including slope factors, *s*). Statistical analyses were done using one-way analysis of variance (ANOVA) with Dunnett's post hoc testing for more than two groups and an unpaired Student's *t*-test for two groups. Superscript letters k, b and t denote significant differences; single letter: $p<0.05$; two letters: $p<0.0001$; k, kk: significantly different from Kv4.3S alone (KChIP effect, binary); b, bb: significantly different from 1a co-expression (special feature of the 1b splice variant); t,tt: significantly different from Kv4.3S + DPP (KChIP effect in the presence of DPP, ternary).

Table S4. Functional characterization of Kv4.3L in the absence and presence of KChIP1 splice variants 1a and 1b in different channel configurations.

<table>
<tr><th>Kv4.3L configuration
(ng cRNA, day)</th><th>$\tau_{1,inact}$
(ms)</th><th>$amp_{1,inact}$
(%)</th><th>$\tau_{2,inact}$
(ms)</th><th>$amp_{2,inact}$
(%)</th><th>$\tau_{3,inact}$
(ms)</th><th>$amp_{3,inact}$
(%)</th><th>$\tau_{1,rec}$
(ms)</th><th>$amp_{1,rec}$
(%)</th><th>$\tau_{2,rec}$
(ms)</th><th>$amp_{2,rec}$
(%)</th><th>$V_{1/2\ act}$
(mV)</th><th>s_{act}
(mV)</th><th>$V_{1/2\ inact}$
(mV)</th><th>s_{inact}
(mV)</th></tr>
<tr><td>Kv4.3L alone
(1.7, d2)</td><td>45.6 ± 10.0
(25)</td><td>72 ± 3
(25)</td><td>145 ± 29.3
(25)</td><td>20 ± 3
(25)</td><td>892 ± 83.0
(25)</td><td>8 ± 1
(25)</td><td>448 ± 156
(6)</td><td>76 ± 14
(6)</td><td>2294 ± 905
(6)</td><td>24 ± 14
(6)</td><td>-4.1 ± 3.1
(9)</td><td>27.8 ± 1.7
(9)</td><td>-66.0 ± 3.7
(9)</td><td>4.4 ± 0.4
(9)</td></tr>
<tr><td>Kv4.3L + 1a
(1.7+10, d2)</td><td>43.6 ± 8.7
(20)</td><td>37 ± 12^{kk}
(20)</td><td>106 ± 16.4^{kk}
(20)</td><td>57 ± 13^{kk}
(20)</td><td>509 ± 57.3^{kk}
(20)</td><td>6 ± 2^{kk}
(20)</td><td>189 ± 31.5^{kk}
(10)</td><td>90 ± 3^{k}
(10)</td><td>4848 ± 1085^{k}
(10)</td><td>10 ± 3^{k}
(10)</td><td>-9.2 ± 4.1^{k}
(10)</td><td>24.9 ± 3.7
(10)</td><td>-60.6 ± 4.5^{k}
(11)</td><td>4.1 ± 0.8
(11)</td></tr>
<tr><td>Kv4.3L + 1b
(1.7+10, d2)</td><td rowspan="2">24.3 ± 4.0^{kk,bb}
(22)</td><td rowspan="2">39 ± 6^{kk}
(22)</td><td rowspan="2">105 ± 13.2^{kk}
(22)</td><td rowspan="2">51 ± 7^{kk}
(22)</td><td rowspan="2">406 ± 50.1^{kk,bb}
(22)</td><td rowspan="2">10 ± 2^{kk,bb}
(22)</td><td rowspan="2">363 ± 60.3^{bb}
(8)</td><td rowspan="2">76 ± 4^{bb}
(8)</td><td rowspan="2">10158 ± 2237^{kk,bb}
(8)</td><td rowspan="2">24 ± 4^{bb}
(8)</td><td rowspan="2">-7.6 ± 3.5
(9)</td><td rowspan="2">25.9 ± 2.0
(9)</td><td rowspan="2">-66.1 ± 4.6^{b}
(9)</td><td rowspan="2">5.1 ± 1.0^{b}
(9)</td></tr>
<tr><td>Kv4.3L + 1b
(1.7+10, d3)</td></tr>
<tr><td>Kv4.3L + DPP
(0.8+2.5, d2)</td><td rowspan="2">39.0 ± 7.7
(5)</td><td rowspan="2">76 ± 2
(5)</td><td rowspan="2">142 ± 26.3
(5)</td><td rowspan="2">14 ± 2
(5)</td><td rowspan="2">840 ± 60.7
(5)</td><td rowspan="2">10 ± 1
(5)</td><td>253 ± 5.2
(3)</td><td>80 ± 2
(3)</td><td>912 ± 28.3
(3)</td><td>20 ± 2
(3)</td><td rowspan="2">-9.2 ± 5.9
(4)</td><td rowspan="2">28.3 ± 2.2
(4)</td><td rowspan="2">-67.4 ± 1.9
(5)</td><td rowspan="2">4.8 ± 0.6
(5)</td></tr>
<tr><td>Kv4.3L + DPP
(0.8+2.5, d3)</td><td colspan="4">-</td></tr>
<tr><td>Kv4.3L + DPP + 1a
(0.8+2.5+5, d2)</td><td rowspan="2">47.3 ± 16.3
(5)</td><td rowspan="2">70 ± 30
(5)</td><td rowspan="2">166 ± 43.5
(5)</td><td rowspan="2">26 ± 27
(5)</td><td rowspan="2">551 ± 81.3^{tt}
(5)</td><td rowspan="2">4 ± 3^{t}
(5)</td><td rowspan="2">46.7 ± 11.4^{tt}
(6)</td><td rowspan="2">94 ± 2^{t}
(6)</td><td rowspan="2">1709 ± 1193
(6)</td><td rowspan="2">6 ± 2^{t}
(6)</td><td rowspan="2">-17.6 ± 5.4^{t}
(6)</td><td rowspan="2">22.7 ± 1.8^{tt}
(6)</td><td rowspan="2">-60.3 ± 3.4^{t}
(6)</td><td rowspan="2">4.4 ± 0.8
(6)</td></tr>
<tr><td>Kv4.3L + DPP + 1a
(0.8+2.5+5, d3)</td></tr>
<tr><td>Kv4.3L + DPP + 1b
(0.8+2.5+5, d2)</td><td rowspan="2">36.0 ± 12.9
(7)</td><td rowspan="2">51 ± 21
(7)</td><td rowspan="2">90.5 ± 27.1^{t,b}
(7)</td><td rowspan="2">44 ± 21^{t}
(7)</td><td rowspan="2">346 ± 65.0^{tt,b}
(7)</td><td rowspan="2">4 ± 1^{tt}
(7)</td><td rowspan="2">110 ± 37.3^{tt,b}
(8)</td><td rowspan="2">74 ± 5^{bb}
(8)</td><td rowspan="2">2482 ± 1098
(8)</td><td rowspan="2">26 ± 5^{bb}
(8)</td><td rowspan="2">-13.2 ± 3.5
(8)</td><td rowspan="2">23.7 ± 0.9^{tt}
(8)</td><td rowspan="2">-68.7 ± 2.8^{b}
(8)</td><td rowspan="2">5.0 ± 0.4
(8)</td></tr>
<tr><td>Kv4.3L + DPP + 1b
(0.8+2.5+5, d3)</td></tr>
</table>

Kv4.3L (long splice variant) was expressed alone or in different channel configurations (i.e., in the absence or presence of DPP) with either KChIP1a (1a) or KChIP1b (1b) in *Xenopus* oocytes (cRNA amounts in ng per oocyte indicated). Functional characterization under two-electrode voltage-clamp was performed on days 1 - 3 after cRNA injection (*d1* - *d3*, as indicated). Mean values ± SD and number of oocytes (n) are given for the time constants of macroscopic inactivation and recovery from inactivation (including relative amplitudes in %), and the voltages of halfmaximal activation and inactivation (including slope factors, *s*). Statistical analyses were done using one-way analysis of variance (ANOVA) with Dunnett's post hoc testing for more than two groups and an unpaired Student's *t*-test for two groups. Superscript letters k, b and t denote significant differences; single letter: $p<0.05$; two letters: $p<0.0001$; k, kk: significantly different from Kv4.3L alone (KChIP effect, binary); b, bb: significantly different from 1a co-expression (special feature of the 1b splice variant); t,tt: significantly different from Kv4.3L + DPP (KChIP effect in the presence of DPP, ternary).

Table S5. Experimental manipulation of Kv4.2 + DPP +1b channel inactivation.

Kv4.2 + DPP + 1b	$\tau_{1,inact}$ (ms)	$amp_{1,inact}$ (%)	$\tau_{2,inact}$ (ms)	$amp_{2,inact}$ (%)	$\tau_{3,inact}$ (ms)	$amp_{3,inact}$ (%)	**$\tau_{1,rec}$ (ms)**	**$amp_{1,rec}$ (%)**	**$\tau_{2,rec}$ (ms)**	**$amp_{2,rec}$ (%)**
wild-type	32.0 ± 3.0<br>(17)	51 ± 3<br>(17)	77.4 ± 6.4<br>(17)	45 ± 3<br>(17)	513 ± 40<br>(17)	4 ± 1<br>(17)	41.4 ± 4.2<br>(8)	57 ± 4<br>(8)	1275 ± 167<br>(8)	43 ± 4<br>(8)
Δ10	32.2 ± 7.1<br>(8)	47 ± 14.5<br>(8)	78.9 ± 14.3<br>(8)	49 ± 12.8<br>(8)	284 ± 74^{mm}<br>(8)	4 ± 2<br>(8)	72.9 ± 15.6^{m}<br>(9)	65 ± 4^{m}<br>(9)	1382 ± 320<br>(9)	35 ± 4^{m}<br>(9)
L400A	30.4 ± 3.5<br>(14)	70 ± 12^{mm}<br>(14)	90.8 ± 20.2<br>(14)	22 ± 10<br>(14)	715 ± 125^{mm}<br>(14)	8 ± 3<br>(14)	51.4 ± 17.6<br>(13)	61 ± 6<br>(13)	1177 ± 265<br>(13)	39 ± 6<br>(13)
N408A	8.3 ± 1.8^{mm}<br>(9)	92 ± 2^{mm}<br>(9)	58.4 ± 29.1<br>(9)	5 ± 3^{mm}<br>(9)	592 ± 98^{m}<br>(9)	3 ± 0.7<br>(9)	690 ± 57<br>(9)	-		
wild-type (2 / **98 K⁺**)	34.9 ± 6.4<br>**19.8 ± 0.8***<br>(5)	88 ± 4<br>**92 ± 3**<br>(5)	136 ± 17.0<br>**88.5 ± 36.3**<br>(5)	12 ± 4<br>**8 ± 3**<br>(5)	-		47.5 ± 5.7<br>**50.8 ± 10.0**<br>(6)	50 ± 3<br>**53 ± 6**<br>(6)	1377 ± 189<br>**568 ± 129****<br>(6)	50 ± 3<br>**47 ± 6**<br>(6)
wild-type (Control / **TEA**)	37.9 ± 4.4<br>**20.3 ± 2.4***<br>(4)	85 ± 1<br>**92 ± 3***<br>(4)	132 ± 16.2<br>**120 ± 40**<br>(4)	15 ± 1<br>**8 ± 3***<br>(4)			48.8 ± 4.4<br>**38.3 ± 3.6***<br>(5)	49 ± 1.1<br>**43 ± 2***<br>(5)	1279 ± 186<br>**1206 ± 398**<br>(5)	51 ± 1<br>**57 ± 2***<br>(5)
Δ10 (2 / **98 K⁺**)	45.0 ± 7.0<br>**26.5 ± 2.2***<br>(6)	78 ± 5<br>**92 ± 5**<br>(6)	138 ± 14.3<br>**102 ± 31.4***<br>(6)	22 ± 5<br>**8 ± 5**<br>(6)			72.7 ± 16.6<br>**72.9 ± 13.8**<br>(6)	65 ± 4<br>**68 ± 7**<br>(6)	1396 ± 339<br>**671 ± 209***<br>(6)	35 ± 4<br>**32 ± 4**<br>(6)
Δ10 (Control / **TEA**)	45.8 ± 8.7<br>**25.9 ± 5.0***<br>(5)	75 ± 3<br>**87 ± 3***<br>(5)	136 ± 18.3<br>**93.7 ± 15.6***<br>(5)	25 ± 3<br>**13 ± 3***<br>(5)			79.4 ± 11.0<br>**75.8 ± 12.4**<br>(6)	64 ± 3<br>**63 ± 3**<br>(6)	1493 ± 248<br>**1303 ± 275***<br>(6)	36 ± 3<br>**37 ± 3**<br>(6)
L400A (2 / **98 K⁺**)	39.6 ± 7.8<br>**24.7 ± 2.4***<br>(8)	89 ± 3<br>**91 ± 2***<br>(8)	532 ± 145<br>**350 ± 88***<br>(8)	11 ± 3<br>**9 ± 2***<br>(8)			57.0 ± 18.7<br>**54.8 ± 7.4**<br>(9)	58 ± 5<br>**71 ± 7***<br>(9)	1254 ± 266<br>**883 ± 263***<br>(9)	42 ± 5<br>**29 ± 7***<br>(9)
N408A (2 / **98 K⁺**)	8.4 ± 0.9<br>**6.7 ± 1.2***<br>(7)	96 ± 0.8<br>**94 ± 2***<br>(7)	355 ± 0.9<br>**224 ± 124**<br>(7)	4 ± 0.8<br>**6 ± 2***<br>(7)			666 ± 63<br>**310 ± 27***<br>(5)	-		

Kv4.2 + DPP + 1b channels were expressed in *Xenopus* oocytes, and functional characterization with the two-electrode voltage-clamp technique was performed under different conditions. Mean values ± SD and number of oocytes (n) are given for the time constants of macroscopic inactivation and recovery from inactivation (including relative amplitudes in %; control groups in normal ND96 solution shown in grey). Statistical analyses were done using one-way analysis of variance (ANOVA) with Dunnett's post hoc testing for more than two groups and an unpaired or a paired Student's *t*-test for comparisons between two groups; significance of mutant effects indicated as m ($p<0.05$) or mm ($p<0.0001$); significant effects of switching to elevated external K^+ or to TEA (bold) indicated as *($p<0.05$).
